# ANRIL/CDKN2B-AS shows two-stage clade-specific evolution and becomes conserved after transposon insertions in simians

**DOI:** 10.1186/1471-2148-13-247

**Published:** 2013-11-13

**Authors:** Sha He, Weiling Gu, Yize Li, Hao Zhu

**Affiliations:** 1 Bioinformatics Section, School of Basic Medical Sciences, Southern Medical University, Shatai Road, Guangzhou 510515, China

## Abstract

**Background:**

Many long non-coding RNA (lncRNA) genes identified in mammals have multiple exons and functional domains, allowing them to bind to polycomb proteins, DNA methyltransferases, and specific DNA sequences to regulate genome methylation. Little is known about the origin and evolution of lncRNAs. ANRIL/CDKN2B-AS consists of 19 exons on human chromosome 9p21 and regulates the expression of three cyclin-dependent kinase inhibitors (CDKN2A/ARF/CDKN2B).

**Results:**

ANRIL/CDKN2B-AS originated in placental mammals, obtained additional exons during mammalian evolution but gradually lost them during rodent evolution, and reached 19 exons only in simians. ANRIL lacks splicing signals in mammals. In simians, multiple transposons were inserted and transformed into exons of the ANRIL gene, after which ANRIL became highly conserved. A further survey reveals that multiple transposons exist in many lncRNAs.

**Conclusions:**

ANRIL shows a two-stage, clade-specific evolutionary process and is fully developed only in simians. The domestication of multiple transposons indicates an impressive pattern of “evolutionary tinkering” and is likely to be important for ANRIL’s structure and function. The evolution of lncRNAs and that of transposons may be highly co-opted in primates. Many lncRNAs may be functional only in simians.

## Background

X-chromosome inactivation in female placental mammals, which causes the products of genes on the X chromosome to have equal dosages in males and females, is controlled by a set of long non-coding RNA (lncRNA) genes, including Xist, Tsix, Jpx, and Tsx (reviewed in [1,2]). The imprinted expression of some mammalian genes is also controlled by lncRNAs (reviewed in [3,4]). In addition to X-inactivation and imprinted gene expression, tissue-specific DNA and chromatin methylation occurs widely in animal somatic cells because in differentiated cells, most genes are silenced by DNA and chromatin methylation. Because mammalian genomes contain only a few genes encoding DNA methyltransferases and polycomb repressive complexes for DNA and chromatin methylation and because these proteins lack sequence-specific DNA-binding subunits, how these proteins are guided to different genomic sites has long been a puzzle. Recently, more than 10,000 lncRNAs have been identified in humans [5-7], and many can bind to both polycomb proteins/DNA methyltransferases and specific DNA sequences [8,9]. As scaffolds bridging protein-DNA interactions, lncRNAs are key players in dynamic and tissue-specific genome modification [10] (recently reviewed in [11,12]). Because dysregulated genome modification can cause diverse diseases, especially cancers, lncRNAs have triggered immense research interest in multiple fields. While lncRNAs are thought to be associated with X-chromosome inactivation and imprinted gene expression in mammals and most lncRNAs were first identified in humans [13], lncRNAs have also been identified recently in multiple non-mammalian organisms [14-16]. Thus, like microRNAs, lncRNAs may be clade- or species-specific, although the details of these patterns remain unknown.

Based upon their locations, lncRNAs can be classified into two groups. Many lncRNAs are located antisense to the genes they regulate; typical examples include AIRN, H19, and Kcnq1ot1, which control the imprinted expression of Igf2, Igf2r, and Kcnq1 [17,18]. Many other lncRNAs are located far from their target genes; for example, HOTAIR lies between HOXC11 and HOXC12 and regulates the expression of HOXD genes in humans [19]. It is relatively easy to experimentally identify antisense lncRNAs; nevertheless, few in-depth analyses have been performed.

The recently identified ANRIL/CDKN2B-AS on human chromosome 9p21 is antisense to and regulates the expression of three cyclin-dependent kinase (Cdk) inhibitors: CDKN2A (INK4a/p16), ARF (p14), and CDKN2B (INK4b/p15) [20]. The expression of ANRIL can induce CDKN2B silencing *in cis* and *in trans* through heterochromatin formation [21]. The silencing of CDKN2B occurs via PRC2 recruitment by ANRIL, which makes the CDKN2B locus H3K27-trimethylated. Depletion of ANRIL increases the expression of CDKN2B [22]. The vital roles of these Cdk inhibitors in cell-cycle control make ANRIL an important molecule in multiple cancers. It is estimated that the genomic region containing ANRIL and CDKN2A/ARF/CDKN2B is altered in 30–40% of human tumours [22]. Understanding the origin and evolution of ANRIL will help to clarify its functions and decipher the evolution of many other lncRNAs.

Studies of lncRNA evolution face three challenges. First, most protein-coding genes in mammals were generated by genome and chromosome duplications that immediately produced all of the gene’s exons. Generated after two rounds of whole-genome duplication [23], lncRNAs are relatively young, and little is known about how and when they obtained multiple exons. Second, like other ncRNAs, lncRNAs exhibit conserved structures but divergent sequences due to compensatory mutations [7]. Thus, specific genome-search techniques that are more powerful than BLAST/BLAT are needed to search for homologous lncRNA genes in different organisms. Third, lncRNAs may be clade- or even species-specific. When a particular lncRNA is absent in a given organism, it may be difficult to determine whether the gene was never present or underwent a birth-and-death process.

The ANRIL gene is not only important but also quite unusual in that it contains 19 exons, making its origin and evolution particularly intriguing. To decipher its evolutionary history, we searched the genomes of 27 organisms, including non-mammalian vertebrates (hereafter called vertebrates), non-placental mammals, non-primate placental mammals (hereafter called mammals), and primates, to obtain sequences homologous to the exons of the human ANRIL gene. In-depth analyses of these sequences yielded several interesting conclusions. ANRIL originated in the eutherian ancestor and initially contained only a few exons and splicing signals. Later, it underwent clade-specific evolution, obtaining additional exons in some mammals but gradually losing exons in rodents. Notably, its genomic sequence expanded significantly in simians (here represented by the marmoset) through the insertion of multiple transposons. Some transposons were inserted into selective sites within the exons, and some transposons were transformed into the exons. These transposons not only modified the sequence and structure of ANRIL but also caused the gene to become highly conserved. A large-scale survey of lncRNAs in the database http://www.lncRNAdb.org reveals that many lncRNAs contain transposons. These findings indicate for the first time that transposons have contributed significantly to lncRNA evolution in simians. This phenomenon is a remarkable aspect of lncRNA evolution in primates.

## Results

### Infernal searches identify putative ANRIL exons only in placental mammals

**Figure 1:**
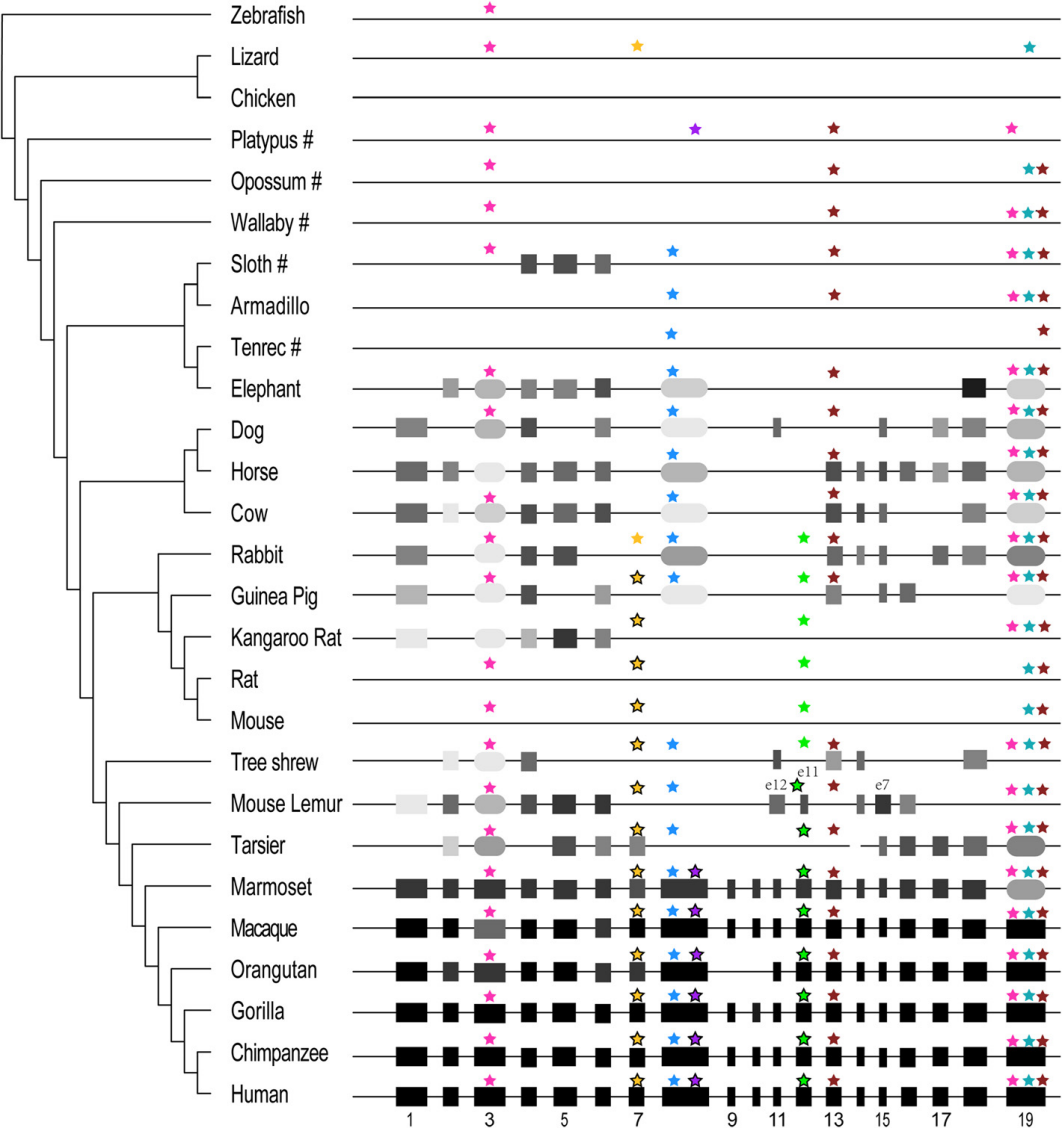
**The ANRIL gene and related potential transposons in vertebrates, mammals, and primates.** The phylogenetic tree for the 27 organisms was adapted from the UCSC Genome Browser. The Infernal search was applied to the whole genome for five organisms whose genomes were not fully assembled (indicated by #) and to the chromosome containing CDKN2A/CDKN2B for the remaining organisms. Exons are indicated by square and rounded boxes (odd-numbered exons are labelled at the bottom). Transposons are indicated by asterisks in different colours (if a transposon occurs in > 500 Infernal hits in a chromosome, the asterisk has a black border). A square box with asterisk(s) above it indicates that the exon contains the transposon(s). A rounded box with asterisk(s) above it indicates that the exon does not contain the transposon(s), but the transposon(s) occur elsewhere in the chromosome. A square box without asterisks indicates that the exon does not contain transposons in all species. A rounded box without asterisks indicates that the exon does not contain the transposon(s), nor do the transposon(s) occur elsewhere in the chromosome. Asterisks without boxes indicate that the transposons were identified but the exons were not. The box length and shading indicate the length and score (reported by Infernal) of each exon. Distances between exons are not shown to scale.

Because lncRNAs exhibit conserved structures but divergent sequences, we used Infernal, a structure-based RNA search program [24], to search for orthologous sequences of ANRIL exons in the genomes of 27 organisms (Figure 1). First, we used Infernal to build 19 covariance models (hereafter called CMs, each containing the structure and sequence information of an exon) based on exons 1 to 19 of the human ANRIL gene. We then searched these CMs against the genomes of selected primates and mammals and found that for multiple CMs, high-scoring hits were obtained only in simians. To make the CMs more representative, we re-built CM1 to CM19 based on the exons in human and the putative exons in macaque and used these CMs to search the 27 genomes (including the macaque genome). The macaque genome was chosen because it is neither so close to the human genome that the resulting CMs would be overly specific nor so distant from the human genome that the structure and sequence information contained in human exons would be weakened or blurred, thus ensuring that truly putative exons could be identified in the target genomes. Still, Infernal searches failed to identify hits of ≥ 3 successive CMs with high or medium scores in vertebrates and non-placental mammals. Sequences homologous to the exons of the human ANRIL gene (hereafter called ‘exons’) first appeared in Xenarthra (sloth) and Afrotheria (elephant) before occurring more widely in Laurasiatheria (dog, horse, and cow). However, in the branch of the species tree comprising rabbit (Lagomorpha) and rodents, the number of exons decreased steadily from rabbit to kangaroo rat; neither medium-scoring hits nor hits of successive CMs were found in mouse or rat. Notably, all 19 exons of ANRIL were present only in simians (Figure 1). Thus, ANRIL may present an impressive case of two-stage, clade-specific evolution.

Our genome search results were carefully verified to ensure their reasonability and reliability. First, we used BLAT at the UCSC Genome Browser to search the human ANRIL exons against the 27 organismal genomes. In mammals and prosimians (and even in marmoset, a simian), the hits were much shorter than the human ANRIL exons, and successive series of putative exons were not found. On the other hand, many hits appeared to be false positives (Additional file 1). In comparison, Infernal produced fewer and longer hits, with two important features: (1) the highest-scoring hits matched the full CMs in most simians and some mammals, had extremely low E-values, and were successively distributed on the DNA strand antisense to CDKN2A/CDKN2B; (2) other hits that did not match the CMs well, with medium or low scores, had very large E-values (Additional file 1). This comparison indicates that, as reported previously [25], Infernal significantly outperformed BLAT in reliably identifying sequences orthologous to ANRIL exons. Second, to confirm that the failure to detect exons in non-placental mammals and vertebrates was not due to the evolutionary distance between human/macaque and these organisms, we built CMs based on the identified exons in rabbit and horse and re-searched these CMs against the genomes of opossum and chicken. Again, no exons were detected. Third, we examined the MultiZ-aligned regions of the 19 exons in the 27 organisms in the UCSC Genome Browser and found that no or only extremely poorly aligned sequences were present in the vertebrates and non-placental mammals (Additional file 2: Figure S1). Finally, to confirm that the failure to detect exons in mouse and rat was not influenced by the human/macaque-based CMs, we built CMs based on the identified exons in rabbit and re-searched these CMs against the mouse and rat genomes. Again, neither medium-scoring hits nor hits of successive CMs were obtained.

### Putative ANRIL exons in mammals lack splicing signals

**Figure 2:**
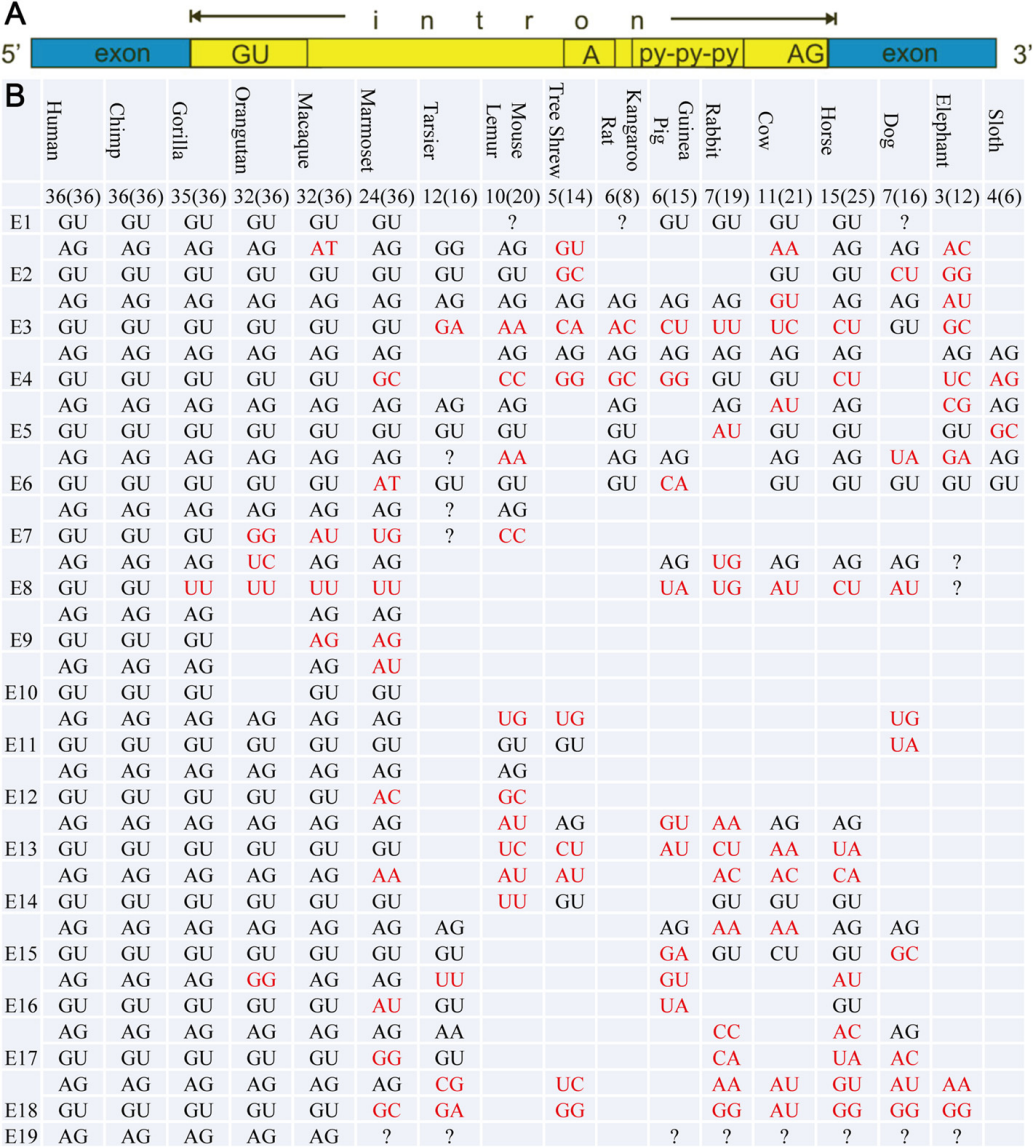
**Splicing signals in ANRIL. (A)** Schematic of splicing sites and signals. **(B)** ANRIL contains significantly more splicing signals in simians than in other mammals and prosimians. Black, red, and ‘?’ indicate conserved, non-conserved, and undetermined splicing signals, respectively. Blanks indicate the absence of exons.

Because the ANRIL gene in mammals has only a few exons, it may not be transcribed and functional. We indirectly addressed this issue by searching for splicing signals across exon-intron boundaries. A recent large-scale study has revealed that the vast majority of lncRNA introns are flanked by canonical splicing signals (GU/AG) and that lncRNA genes do not differ from protein-coding genes in splicing-signal usage [7]. Our search found that in simians, 191 exon-intron boundaries had splicing signals, while 20 did not. In mammals (including the two prosimians, mouse lemur and tarsier), 85 exon-intron boundaries had splicing signals, while 86 did not (Figure 2). The significant difference between simians and other mammals (χ= 76.4539, p-value < 2.2e-16) suggests that mammalian ANRIL genes may not be properly transcribed or may yield only rudimentary transcripts. This result, together with the finding that ANRIL contains only a few exons in mammals, suggests that ANRIL may be functional only in simians.

### Multiple transposons are inserted into ANRIL exons in simians

A surprising finding of our genome search was that some CMs produced abundant hits (e.g., CM7 produced 94335 hits on chromosome 1 in the marmoset genome), strongly suggesting that some ANRIL exons are or contain transposons. We therefore used RepeatMasker with Repbase, an important transposon database, to scan human ANRIL. The scan identified 10 transposons and simple repeats (Table 1). Because this result indicated that transposons might be present in the ANRIL genes of other organisms, the Infernal hits were closely examined to detect potential transposons in the ANRIL exons of multiple organisms. For convenience, hereafter we use TE# to denote abundant hits matching a fixed region within CM# and E#TE# to specifically denote the region within CM#. Numerous hits matched bp 81–266 in CM3 (TE3), bp 202–395 in CM8 (TE8a), bp 396–678 in CM8 (TE8b), bp 30–86 in CM19 (TE19a), bp 249–588 in CM19 (TE19b), and bp 375–457 in CM19 (TE19c) (these TEs, including their positions within the ANRIL exons, are not necessarily identical to the RepeatMasker-identified transposons in human ANRIL). Specifically, TE3 is much longer than the MER1A in human exon 3. Our examination of Infernal hits revealed that some TEs were simian-specific, but many were present in mammals; nearly all of them were inserted or transformed into ANRIL exons at or before the divergence between marmoset and other simians.

**Table 1:** RepeatMasker-identified transposons

**Start position**	**End position**	**Location within human ANRIL**	**Matching repeat**	**Repeat class/family**	**ID**
318	362	Exon 1	(CGGCG)n	Simple repeat	1
791	845	Exon 3	MER1A	DNA/hAT-Charlie	2
1250	1366	Exon 7	AluSx3	SINE/Alu	3
1589	1763	Exon 8	MLT1I	LTR/ERVL-MaLR	4
1772	2083	Exon 8	THE1A	LTR/ERVL-MaLR	5
2327	2436	Exon 12	AluJb	SINE/Alu	6
2447	2527	Exon 13	L2c	LINE/L2	7
3239	3340	Exon 19	MIR3	SINE/MIR	8
3489	3563	Exon 19	(TCA)n	Simple repeat	9
3579	3792	Exon 19	MIR	SINE/MIR	10

In tree shrew (a small mammal of the order Scandentia, closely related to primates) and in two prosimians (mouse lemur and tarsier), the ANRIL exons showed peculiar features: (1) fewer exons were present in these organisms than in Laurasiatheria; (2) in mouse lemur, the positions of exon 12 and exon 7 (both TE-transformed) were reversed; and (3) in tarsier, CM7 and CM12 produced many hits with scores higher than those of exon 7 and exon 12. Moreover, of the 94335 hits for CM7 on chromosome 1 in the marmoset genome, 15000 had higher scores than marmoset exon 7 itself, indicating that these regions may be closer to human exon 7. These findings suggest that the ANRIL gene evolved substantially from mammals to prosimians and from prosimians to simians and that this process was strongly influenced by transposon activity.

### Transposons are inserted into exons within structural contexts

As mobile elements, active transposons insert into many sites in a genome. What drives their insertion and whether transposons selectively insert into specific sites are interesting questions with limited answers. We examined the hits for CM3 and CM8, including TE3, TE8a, and TE8b, across the set of genomes. In rabbit, exon 3 matched bp 1–74 and 307–328 of CM3 but left bp 75–306 of CM3 unmatched. Meanwhile, there is a hit elsewhere that exactly matched bp 75–306 of CM3 (and more hits matched bp 80–265 of CM3). If this hit were inserted into the gap in exon 3, a sequence nearly identical to human exon 3 would be obtained. In the genomes of other non-simian organisms, all exon 3 sequences contained a gap equal to or smaller than that found in rabbit exon 3, and hits matching bp 80–265 of CM3 were found widely elsewhere (especially in zebrafish, lizard, and the three non-placental mammals). Note that compared to these hits (designated TE3), the MER1A transposon in human ANRIL is located closer to the 3′ end of exon 3.

**Figure 3:**
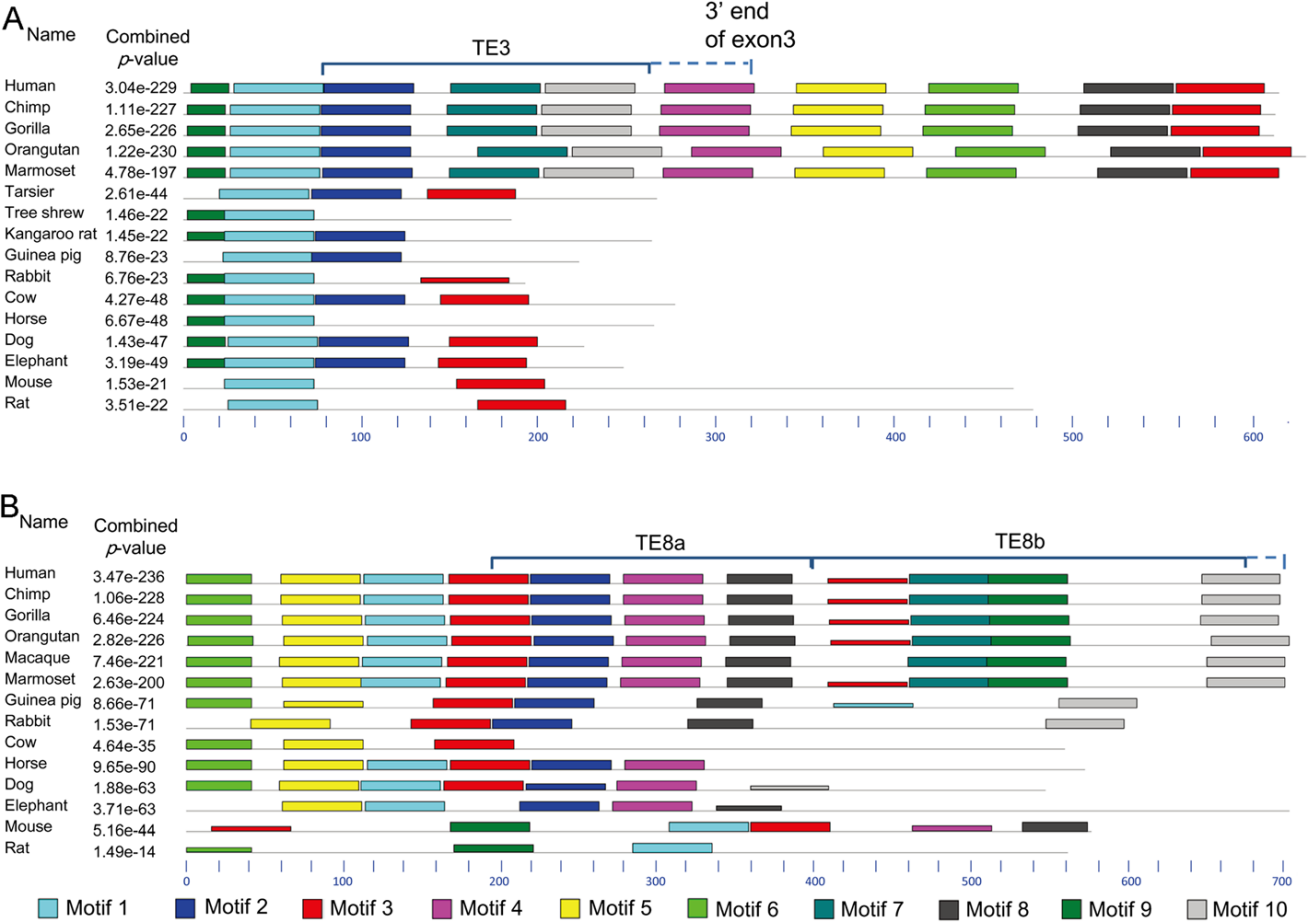
**TE3 and TE8b are inserted within a structural context in simians.** Coloured bars indicate MEME-identified motifs; black lines indicate featureless sequences. The mouse and rat sequences used for motif identification were obtained using BLAT to search human exon 3 and exon 8 against the mouse and rat genomes. **(A)** Four motifs can be identified in mammalian and prosimian exon 3. The sequences covering the first two motifs are highly conserved. TE3 corresponds to bp 80–265 of CM3. The UCSC Genome Browser indicates that in simians, the MEA1A transposon crosses the boundary between exon 3 and intron 3; and a BLAT genome search indicates that the 300 bp at the 5′ end of intron 3 exist only in simians. The insertion of the MEA1A transposon apparently provides the GU splicing signal for exon 3. **(B)** MEME identifies multiple motifs at the TE8b insertion site. TE8a is absent in some mammals, but the gap corresponding to TE8a in these mammals is smaller than TE8a, and these mammalian exon 8 sequences contain several motifs found in TE8a.

To examine the context of the insertion of TE3, we used MEME, a program that detects potential motifs in multiple sequences, to analyse the exon 3 sequences. MEME identified multiple motifs within exon 3 in these organisms. A sequence alignment revealed that the two initial motifs were highly conserved. Currently, we are unable to satisfactorily explain why the sequence of motif 2 (shown in blue) in some mammalian and prosimian exon 3 is highly similar to the sequence of motif 2 (shown in blue) in simian E3TE3. Probably, motif 2 contains conserved residues surrounding the insertion site of E3TE3 and occurs at the position in a species-specific way. To check whether motif 3 (shown in red), which was present in mammalian and prosimian exon 3 sequences but absent in simian exon 3 sequences, was really lost in simians, we ran MEME using exon 3 + 300 bp at the 5′ end of intron 3 in simians. We found that this motif was shifted from exon 3 in mammals and prosimians into intron 3 in simians (Figure 3A). MEME revealed that the insertion of TE8b into exon 8 also occurred in a motif-specific context (Figure 3B).

We further analysed intron 3 and intron 8. In human, the simian-specific MER1A transposon penerates into intron 3. Additionally, the THE1A transposon (TE8b) in human exon 8 was followed by another simian-specific transposon at the 5′ end of intron 8. Thus, simian-specific transposons also provide the GU splicing signals for the matured exon 3 and exon 8 in simians. Compared to the 3′ ends of exon 3 and exon 8 in mammals, the 3′ ends of exon 3 and exon 8 in simians appear more structured.

### TEs have modified the sequences and structures of ANRIL exons

The insertion of multiple TEs into ANRIL should affect the structure and function of this gene. Because an lncRNA could fold into many different structures, making structural prediction difficult, we examined how the insertion of TEs modified the sequences and structures of the ANRIL exons. TE3 exhibits a palindromic structure that is conserved from lizard to human, with two terminal inverted repeats flanking a short internal region (Additional file 2: Figure S2A). This specific structure causes the formation of a stem structure (and a pair of high-scoring hits for CM3 at the same position on the sense and antisense strands in simians) (Additional file 2: Figure S2A). To determine whether the stem structure contains any microRNAs, we searched E3TE3 against the microRNA database (http://www.mirbase.org) and found that it matched the stem-loop structure of two miRNA families with high scores (hsa-mir-645/ptr-mir-645/ppy-mir-645 with score = 140 and E-value = 5e-05; oan-miR-138/aca-miR-138-1 with score = 75 and E-value = 0.62) (Additional file 2: Figure S3).

Next, we examined exon 7, exon 12, and exon 13, which were transformed by TE7, TE12, and TE13. Exon 12 and the highest-scoring TE12 had highly similar sequences, but exon 12 contained a 20-bp sequence in mammals and prosimians that was absent in simians (Additional file 2: Figure S2B). To determine the impact of this 20-bp sequence on the structure of exon 12, we used RNAfold to predict the structures of exon 12 and the highest-scoring TE12. We found that without this 20-bp sequence, exon 12 formed a more stable hairpin (Additional file 2: Figure S2C). In contrast, exon 13 contained a 40-bp sequence that was absent in the highest-scoring TE13 (Additional file 2: Figure S2D). RNAfold revealed that the 40-bp addition caused more nucleotides to pair in exon 13 than in the highest-scoring TE13. Only single-nucleotide differences were found between exon 7 and the highest-scoring TE7, providing no clear evidence of structural effects. These results indicate that multiple TEs may have considerably modified the sequences and structures of the ANRIL exons, but their impact on the global structure of ANRIL remains unclear.

### ANRIL exons became conserved after TE insertions

**Figure 4:**
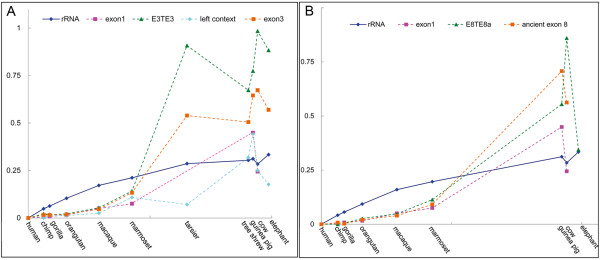
**The insertion of TE3 and TE8b into exon 3 and exon 8 affected the evolution of exon3 and exon 8.** The divergences between human and chimpanzee, gorilla, orangutan, macaque, marmoset, tarsier, three shrew, guinea pig, cow, and elephant are 6.3, 8.8, 15.7, 29.0, 42.6, 65.2, 90.4, 92.3, 94.2, and 98.7 Mya (the species divergence times were acquired from http://www.timetree.org). The displayed are the pairwise sequence distances between human and these species at these time points along the time axis. These pairwise distances indicate that exon 3 and exon 8 became conserved in simians after the insertion of TE3 and TE8b. **(A)** Pairwise distances of the concatenated 12S and 16S mitochondrial rRNAs, exon 1, E3TE3, the left context of the TE3 insertion site, and exon 3. **(B)** Pairwise distances of the concatenated 12S and 16S mitochondrial rRNAs, exon 1, E8TE8a, and the ancient exon 8 (the 5′ end + E8TE8a). All distances were computed using the F84 model in PHYLIP.

Many transposons are suggested to have evolved in a nearly neutral manner [26], but some have played active roles during primate evolution [27]. Thus, we performed further analyses to examine whether TE insertions accelerated or slowed the evolution of ANRIL. The evolution of the concatenated 12S and 16S mitochondrial-rRNA sequences, which have been widely used in phylogenetic studies [28], was used for comparison. First, using the F84 model in PHYLIP, we calculated the pairwise distances of the concatenated rRNAs, exon 1, E3TE3, the left context of the TE3 insertion site, and exon 3 (Figure 4A) and of the concatenated rRNAs, exon 1, E8TE8a, and the ancient exon 8 (the 5′ end + E8TE8a) (Figure 4B) between human and other species. All three exons evolved more rapidly in mammals but less rapidly in simians compared to the two rRNAs. The insertion of TE3 into the ancient exon 3 and of TE8b into the ancient exon 8 may have contributed to the conservation of exon 3 and exon 8 in simians. We also used the Maximum Composite Likelihood model in MEGA to repeat the computation and obtained the same results (Additional file 2: Figure S4).

**Figure 5:**
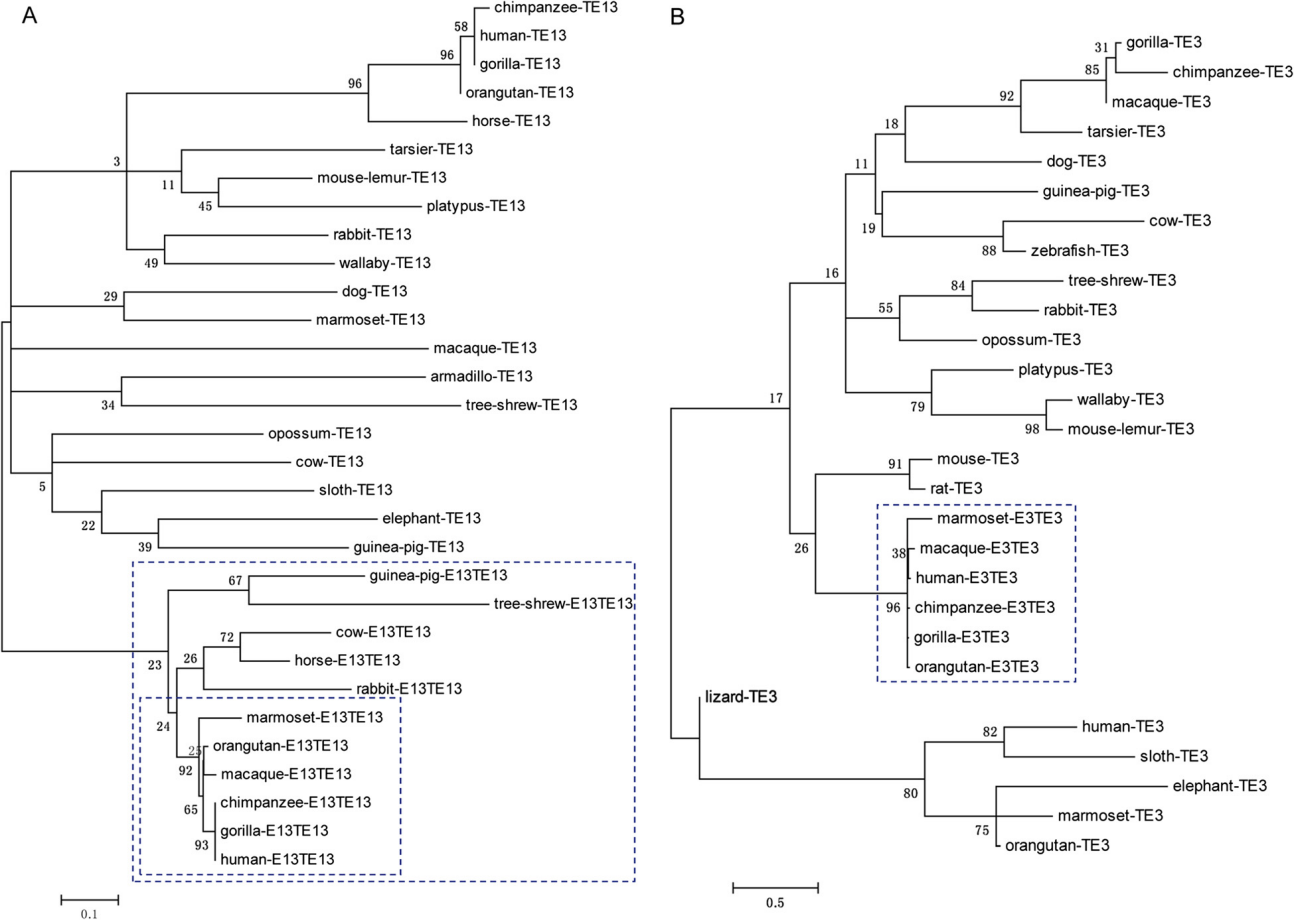
**TEs became conserved after transforming or inserting into ANRIL exons.** MEGA 5.1 was used to predict the most appropriate substitution models and to build ML (maximum likelihood) trees. Both trees were built using the Tamura three-parameter model. Numbers indicate bootstrap values, and the scale at the bottom measures genetic distances in nucleotide substitutions per site. **(A)** ML tree of exon 13 (E13TE13) and TE13 (the highest-scoring free TE13 located outside the ANRIL gene). All E13TE13 sequences are clustered together in a large subtree with a reasonable topology (marked by the large dashed square). In simians, the E13TE13 sequences are not only clustered together but also have short terminal branches (marked by the small dashed square). In contrast, the free TE13 sequences are clustered together in the remaining large subtree with a meaningless topology. A relative-rate test confirms that mammalian TE13 sequences have evolved more rapidly than simian E13TE13 sequences. Thus, the insertion of TE13 into ANRIL (not merely into an ANRIL exon) also constrained the evolution of TE13. **(B)** ML tree of E3TE3 (the TE3 in exon 3) and TE3 (the highest-scoring free TE3 located outside ANRIL). All E3TE3 sequences in simians are not only clustered together but also have short terminal branches. In agreement with Figure 4, this result indicates that TE3 became highly conserved after inserting into exon 3.

Second, to examine whether insertion or transformation into ANRIL exons affected the evolution of the TEs themselves, we built phylogenetic trees for TE13/exon 13 (Figure 5A, Additional file 2: Figure S5A), TE3/E3TE3 (Figure 5B, Additional file 2: Figure S5B), TE7/exon 7 (Additional file 2: Figures S6A and S7A), TE12/exon 12 (Additional file 2: Figures S6B and S7B), and TE8a/E8TE8a (Additional file 2: Figures S6E and S7E) (each TE indicates the highest-scoring independent TE). In these trees, the inserted and transformed TEs (in simians) were grouped together with high statistical support and had short terminal branches, but the free TEs (in all other organisms) were not reliably grouped together and had long terminal branches. Thus, the transposons within the ANRIL gene in simians are most likely vertically inherited from ancestral sequences instead of being copied from elsewhere. Relative rate tests confirmed that the highest-scoring free TE12 evolved faster than exon 12 in simians; more meaningfully, exon 13 in mammals (see Figure 1) evolved faster than exon 13 in simians (the exact probabilities computed by the RRTree method were 0.00177 and 0.01387, respectively) [29]. In the maximum-likelihood and Bayesian trees, the subtrees of exon 13 and E3TE3 were highly consistent, but the remaining parts were not comparable, lending further support to the above conclusion.

### Multiple transposons may have contributed to lncRNA evolution

The above results provide compelling evidence that transposons were essential to the evolution and especially to the maturation of ANRIL. To examine whether the TEs identified in ANRIL also occur in other lncRNAs, we built CMs of these TEs and searched these CMs against the lncRNAs in http://www.lncRNAdb.org[30]. The CMs of E7TE7, E12TE12, and E19TE19c produced medium- or high-scoring hits in multiple lncRNAs (Additional file 2: Table S1). To examine whether transposons generally make important contributions to lncRNAs, we used RepeatMasker to scan all lncRNAs in the lncRNA database. We found that many lncRNAs contain multiple transposons (Table 2). These results suggest that transposons are widely associated with lncRNA evolution.

**Table 2:** LncRNAs containing multiple SINEs and LINEs

**lncRNA**	**SINEs**	**LINEs**
BACE1AS_Human CB960709	2	0
Cyrano_Human ENST00000500949.2	6	0
Emx2os_Human NR002791.2	4	2
Evf2_Human ENST00000430027.2	3	3
lincRNA-p21_Mouse NR_036469.1	2	0
lincRNA-RoR_Human	1	2
NTT_ Human U54776.1	9	5
Otx2os1_Human NR_029385	3	3
PR antisense transcripts_Human	1	2
SNHG3_Human NR_036473.1	5	1
UM9-5_Human NR_002813.1	4	4
Xist_Human NR_001564.1	5	1
Zfhx2as_Human	4	0

## Discussion

Most protein-coding genes in mammals were generated by genome and chromosome duplication [23], but when and how lncRNAs obtained multiple exons remain poorly understood. The answers to these questions will help to decipher when lncRNAs obtained their functions. The lncRNA Xist obtained its exons by the pseudogenisation of protein-coding genes [31], but this phenomenon is unlikely to have occurred widely. With 19 exons, ANRIL provides a valuable case study for lncRNA evolution. Consistent with the hypothesis that most lncRNAs occur in placental mammals [13], we found that ANRIL originated in the eutherian ancestor and gradually obtained more exons during its evolution. The evolution of ANRIL shows several notable features. First, mammalian ANRIL genes lack splicing signals and thus may not be properly transcribed. Second, simian ANRIL genes contain splicing signals and multiple transposons. Third, pairwise sequence distances, phylogenetic trees, and relative-rate tests all indicate that ANRIL became highly conserved in simians after transposon insertion. Fourth, our analysis of intron 3 and intron 8 indicates that simian-specific transposons may also provide splicing signals for ANRIL in simians. Finally, ANRIL apparently gradually lost exons during rodent evolution, containing numerous exons in guinea pig but none in mouse and rat. Although human ANRIL is known to contain transposons [32] and transposons are known to have contributed significantly to human and vertebrate lncRNAs [33,34], this report provides the first in-depth analysis showing that the insertion of multiple transposons into ANRIL in simians may have been essential for the evolution and function of ANRIL. We are now examining whether significant transposon insertion also occurred in other simian lncRNAs. Because lncRNA exons lack a codon structure, it is unclear whether the transformation of transposons into ANRIL exons involved a typical exonisation process [35]. Importantly, ANRIL is the first typical case of clade-specific evolution of lncRNAs, and further studies are needed to elucidate the birth-and-death process in rodents. We postulate that the distinct pattern of two-stage, clade-specific evolution may be a feature of many other lncRNAs.

The reported level of homology between human and mouse H19 (an lncRNA controlling the imprinted expression of Igf2r) is approximately 66%, while that between human and mouse Xist is approximately 49% (but much higher in exon regions) [36]. Because Infernal shows high power to detect orthologous sequences of ANRIL exons in mammals, the absence of high-scoring hits or medium-scoring hits for successive CMs reliably indicates the absence of ANRIL in mouse and rat. Although no ANRIL exons were found in mouse and rat, a large region of the mouse (and human) chromosome 9p21 contains single-nucleotide mutations that are associated with many diseases, including coronary artery disease [37,38]. A recent review has summarised these mutations [39], showing that nearly all of them are located in ANRIL introns. Aligned sequences in the UCSC Genome Browser show that ANRIL introns contain multiple conserved sites or sites with strong epigenomic marks, indicating the functional importance of ANRIL intron sequences.

Some transposons have specifically contributed to mammalian evolution through the rewiring of pregnancy-related gene-regulatory networks [40]. During early primate evolution, many thousands of DNA elements became integrated and fixed [41], and numerous primate-specific SINEs (short interspersed nucleotide elements) triggered the evolution of primate-specific functions [42]. These transposon bursts in primates likely strongly influenced the structural evolution of primate genomes [27], but their functional significance remains inadequately understood. Our analysis of ANRIL reveals that transposons not only inserted or transformed into ANRIL exons but also caused ANRIL to become highly conserved in simians. Thus, these transposons obtained functional significance by contributing to lncRNA evolution, and the evolution of significant lncRNAs and transposons may be deeply co-opted. This postulation can reasonably explain the findings that many lncRNAs contain multiple transposons and that transposons contribute significantly to human and vertebrate lncRNAs [33,34]. Given that approximately one-third of lncRNAs appear to have arisen within the primate lineage [7] and that many transposons are primate-specific, such co-option has likely had a profound impact on primate evolution and physiology [43]. Specifically, the absence of ANRIL in mouse and rat and the truncated and non-functional HOTAIR in mouse may make the epigenomic regulation of many important genes differ between humans and mice/rats [44,45]. Transposons also influence genome methylation at many sites in somatic cells. These observations raise a critical question: to what extent do lncRNAs and transposons affect the comparability of human and mouse/rat cancers?

François Jacob postulated that the emergence of novel forms and functions over time can occur via a ‘tinkering’ process through random combinations of pre-existing elements [46]. More recently, it has been suggested that the pool of transposable elements can be domesticated to serve as a ‘warehouse’ for natural selection, potentially acting as a source of lineage-specific elements [47,48]. Cordaux *et al*. explored an example of tinkering along the human evolutionary lineage and identified a primate-specific chimeric gene consisting of a host gene merged with a transposable element [49]. Subsequent reports have further examined such phenomena [50,51]. ANRIL provides a remarkable example because it involves the domestication of both new and ancient transposons with impressive site selectivity. Our analyses of ANRIL also raise the intriguing question of the fate of the transposons in tree shrew and prosimians. Our results should further promote the recently renewed interest in transposon function and evolution [52-54].

## Conclusions

Transposons contribute significantly to the evolution of ANRIL and considerable other lncRNAs. ANRIL obtained splicing signals and became conserved after transposon insertions in simians but lost all exons in some rodents, showing a two-stage and clade-specific evolutionary process.

## Methods

### Identifying sequences homologous to exons in the ANRIL gene

First, RNAfold (http://rna.tbi.univie.ac.at/) was used with the default parameters to predict the structures of the 19 exons in human ANRIL (NR_003529). Based on the predicted structures, Infernal v1.0.1 was used with the default parameters to build 19 covariance models (CMs) and to search the 19 CMs against the macaque genome [24]. Second, LocARNA + RNAalifold (http://rna.tbi.univie.ac.at/) were used with the default parameters to align the 19 exons of human and macaque ANRIL and to predict the structures of the 19 aligned exons. Based on these predicted structures, Infernal was used to re-build 19 new CMs. Third, these CMs were used to search the genomes of 27 organisms. To confirm that the failure to detect exons in mouse and rat was not influenced by the use of CMs based on two primates, we built CMs based on the identified exons in rabbit and used these CMs to re-search the mouse and rat genomes. To confirm that the failure to detect exons in non-placental mammals and vertebrates was not influenced by the use of CMs based on the primates, we built CMs based on the identified exons in rabbit and horse and used these CMs to re-search the opossum and chicken genomes. The following unmasked genomic sequences were downloaded from http://www.ensembl.org for the genome search: human (GRCh37.57), chimpanzee (CHIMP2.1.57), gorilla (gorGor3.1.64), rhesus macaque (MMUL_1.57), orangutan (PPYG2.64), marmoset (C_jacchus3.2.1.62), tarsier (tarSyr1.53), tree shrew (TREESHREW.50), lemur (micMur1.48), cow (Btau_4.0.57), horse (EquCab2.57), dog (BROADD2.57), kangaroo rat (dipOrd1.53), mouse (NCBIM37.57), rat (RGSC3.4.57), guinea pig (cavPor3), rabbit (oryCun2.64), armadillo (dasNov2.54), sloth (choHof1.63), elephant (loxAfr3), tenrec (TENREC.50), wallaby (Meug_1.0.55), opossum (BROADO5.50), platypus (ornAna1), zebrafish (Zv9.60), chicken (WASHUC2.54), and lizard (AnoCar2.0.64). Sequences of the ANRIL gene in simians are given in Additional file 3.

### Identifying transposons in human ANRIL and in lncRNAs in the lncRNA database

RepeatMasker (http://www.repeatmasker.org/cgi-bin/WEBRepeatMasker) and the database Repbase were used with the default parameters to identify transposons in the human ANRIL gene, in the Infernal search hits, and in lncRNAs in the http://www.lncRNAdb.org database [30].

### Identifying motifs in ANRIL exons

MEME, a program that identifies potential motifs in multiple sequences, was used to identify motifs within the exons of the ANRIL gene. The default parameters were used, except that ‘motif number’ was set to 10 [55].

### Sequence alignment and phylogenetic analysis

MAFFT and LocARNA were used with the default parameters to align the transposons and exons [56]. MEGA v5.1 and PHYLIP v3.69 were used with the default parameters to calculate the pairwise distances of transposons and exons between organisms based on the maximum composite likelihood and F84 models [57,58]. The divergence times between organisms were acquired from http://www.timetree.org[59]. MEGA v5.1 was used to find the most appropriate substitution models and to build maximum likelihood (ML) trees for the transposons and exons. The default parameters were used, except that 1000 bootstrap replications were performed. MrBayes was used with the default parameters to build Bayesian trees for the transposons and exons [60]. Relative-rate tests were performed using RRTree [29].

## Competing interests

The authors declare that they have no competing interests.

## Authors’ contributions

HZ conceived and designed the research. HZ, WG, and YL searched the genomes. YL searched the lncRNA database. HZ and SH analysed the data. HZ wrote the paper. All authors read and approved the final manuscript.

## Supplementary Material

Additional file 1 The Infernal and BLAT search results. Click here for file

Additional file 2 The supplementary figures and tables. Click here for file

Additional file 3 ANRIL sequences in primates. Click here for file
