# Supplementary material for: ANRIL/CDKN2B-AS shows two-stage clade-specific evolution and becomes conserved after transposon insertions in simians
Source: BMC Evol Biol. 2013 Nov 13;13:247. doi: 10.1186/1471-2148-13-247 (PMC3831594; doi:10.1186/1471-2148-13-247)
Supplement: Additional file 1 — The Infernal and BLAT search results. [file 1471-2148-13-247-S1.pdf]

Supplementary file 1 - Blat/Infernal search results. Large numbers in "chr#" and "strand" are scaffold/contig numbers and CHRO numbers, respectively. Empty indicates no result. Only the highest- and second highest-scoring hits are displayed

				Blat search results			Infernal search results				
**species**	**exon**	**span**	**chr**	**strand**	**score**	**identity**	**span**	**chr #**	**strand**	**score**	**E-value**
**Platypus**	E1=371	75580	4	-	36	97.4					
		47	Cotig5544	+	29	94					
	E2=162	20	X5	+	20	100					
		20	Cotig80000	+	20	100					
	E3=328										
	E4=135	25	Ultra143	+	23	100					
		22	Ultra524	-	22	100					
	E5=150	37	7	+	26	100					
		371	Cotig15811	-	24	100					
	E6=123	25	Cotig7463	-	25	100					
	E7=136	110	Cotig105522	-	79	87.1					
		95	Cotig162904	-	67	85.3					
	E8=700	317	Cotig204310	+	212	88.9					
		34	11	+	28	81.3					
	E9=74	20	7	+	20	100					
	E10=75										
	E11=96										
	E12=119	98	7	+	32	97.2					
	E13=143	20	Ultra483	-	20	100					
		20	Ultra103	-	20	100					
	E14=113	491	Ultra225	-	27	96.6					
		27	Ultra56	-	24	100					
	E15=76	21	1	+	21	100					
	E16=134	20	Cotig2774	-	20	100					
	E17=126	43	1	+	28	96.8					
		40	10	-	27	96.6					

	E18=211	207	3	+	25	100					
		32	Cotig8167	-	24	87.5					
	E19=596	130	6	+	65	97.2					
		252	Ultra9	-	62	97.2					
**Opossum**	E1=371	27	3	-	25	96.3					
		28	2	+	24	80.8					
	E2=162	267	6	-	27	100					
		26	6	-	24	100					
	E3=328	39	2	-	25	96.3					
		22	U	-	22	100					
	E4=135	37	4	-	27	87.1					
		33	2	-	27	76.7					
	E5=150	41	1	-	26	100					
		44	3	-	25	100					
	E6=123	21	1	-	21	100					
		20	5	+	20	100					
	E7=136	46	5	-	28	80.5					
		35	3	+	28	77.2					
	E8=700	28	1	-	22	95.9					
		24	1	+	22	95.9					
	E9=74	31	6	-	23	100					
		21	8	+	21	100					
	E10=75	34	7	-	24	96.2					
	E11=96	24	4	+	20	91.7					
	E12=119										
	E13=143	26	2	+	22	100					
		22	4	-	20	95.5					
	E14=113	20	4	-	20	100					
		22	3	-	20	95.5					
	E15=76										
	E16=134	26	6	-	23	84.7					
		20	7	+	20	100					
	E17=126	20	4	+	20	100					
	E18=211	224784	3	+	36	100					
		28	8	+	24	92.9					

	E19=596	261	2	+	68	97.4					
		72	2	+	59	87					
**Wallaby**	E1=371	31	GL103889	+	27	96.7					
		25	GL104774	+	24	80					
	E2=162	20	GL111150	-	20	100					
	E3=328	29	GL098381	-	28	86.3					
		26	GL080124	+	24	96.2					
	E4=135	31	GL048611	+	28	74.2					
		33	GL102068	-	27	96.7					
	E5=150	121	GL068143	-	29	100					
		39	GL084111	-	25	96.3					
	E6=123										
	E7=136	31	GL062437	-	25	90.4					
		24	GL129393	+	23	79.2					
	E8=700	28	GL065110	+	27	67.9					
		29	GL069634	+	24	100					
	E9=74	23	GL092770	+	21	100					
		20	GL134166	+	20	100					
	E10=75										
	E11=96	45	GL098888	-	30	94					
	E12=119	34	GL097307	+	26	88.3					
		29	GL110911	-	21	86.3					
	E13=143	20	GL087131	+	20	100					
	E14=113	33	GL105079	-	23	96.2					
		24	GL114816	+	23	87.5					
	E15=76	20	GL069973	-	20	100					
	E16=134	22	GL095742	+	22	100					
		20	GL056132	+	20	100					
	E17=126	57	GL156609	-	31	97.2					
		32	ABQO011173872	-	27	96.6					
	E18=211	32	GL062535	-	24	100					
		41	GL105147	-	23	96.2					
	E19=596	70	GL105239	-	62	82.9					
		63	GL102360	-	57	82.6					

**Sloth**	E1=371	34	106431	+	25	96.3					
		26	28297	+	23	80					
	E2=162	20	96569	+	20	100					
		20	91030	+	20	100					
	E3=328	33	41458	-	27	96.7					
		31	1356	+	27	96.7					
	E4=135	35	26800	+	27	89.7	1-135	scaffold_21706	+	66.67	7.26E-11
		42	57669	-	25	93.2	1-135	scaffold_26209	-	35.35	0.00161
	E5=150	66	30677	-	36	92.7	1-150	scaffold_21706	+	95.31	1.59E-16
		21	64332	-	21	100	1-150	scaffold_110619	-	28.51	0.1369
	E6=123	39	21706	+	35	84.7	1-123	scaffold_21706	+	58.61	1.12E-11
							1-123	scaffold_66627	-	28.39	0.002994
	E7=136	20	14210	-	20	100					
	E8=700	46	192702	-	40	93.5					
		99	88065	+	40	93.5					
	E9=74	33	20333	+	24	96.2					
		23	39741	+	21	95.7					
	E10=75										
	E11=96										
	E12=119	286	70656	+	45	91					
		21	394539	+	21	100					
	E13=143										
	E14=113	45	260644	-	25	100					
		42	284239	+	25	100					
	E15=76	20	7701	+	20	100					
	E16=134										
	E17=126	292	3223	+	28	93.8					
		22	24224	-	20	95.5					
	E18=211	26	5481	-	22	95.9					
		21	206403	-	21	100					
	E19=596	131	478	-	60	74.7					
		179	23814	+	55	93.9					
**Armadillo**	E1=371	277	JH566723	+	173	85.4					
		20	JH577683	+	20	100					

	E2=162	36	JH564267	+	25	100					
		31	JH570143	-	24	96.3					
	E3=328	37	JH575964	-	28	93.6					
		28	JH566673	-	22	95.9					
	E4=135	38	JH567186	+	29	96.8					
		15118	JH570430	-	28	100					
	E5=150	129	JH566723	+	73	79.4					
		26	JH569432	-	22	96					
	E6=123	20	JH577068	+	20	100					
	E7=136										
	E8=700	93	JH562329	+	45	78.5					
		46	JH569587	+	43	91.4					
	E9=74	22	JH577761	-	22	100					
		23	JH570793	-	21	95.7					
	E10=75										
	E11=96	65	JH566723	+	55	92.4					
		27	JH569903	+	24	77					
	E12=119	20	JH575781	-	20	100					
	E13=143	25	JH569502	+	24	84					
		36	JH569526	-	23	100					
	E14=113	21	JH582994	+	21	100					
		21	JH575483	+	21	100					
	E15=76	151	JH583683	-	26	96.6					
		26	JH570731	+	24	96.2					
	E16=134	24	JH574710	+	23	87.5					
		21	JH577154	+	21	100					
	E17=126	20	JH575723	-	20	100					
	E18=211	22	JH581209	-	22	100					
		20	JH575150	-	20	100					
	E19=596	65	JH574046	+	56	88.6					
		60	JH562375	+	54	81.4					
**Tenrec**	E1=371	28	181397	+	27	82.2					
		29	286342	-	26	96.5					
	E2=162	26	176182	-	23	88					
		26	90489	+	23	88					

	E3=328	30	277125	-	25	72.5					
		192	313126	+	25	100					
	E4=135	30	239160	-	25	96.3					
		280	319640	+	25	100					
	E5=150	38	322685	+	27	93.8					
		26	282186	-	25	65.4					
	E6=123	35	180947	+	25	92.9					
	E7=136	66	150496	+	31	97					
		31	123496	-	27	93.6					
	E8=700	23	277442	+	23	100					
		22	207871	+	22	100					
	E9=74	23	276808	+	21	100					
		23	184850	+	21	100					
	E10=75	25	218417	-	23	96					
	E11=96										
	E12=119	275	314882	-	40	90					
		25	284873	+	23	100					
	E13=143	31	289466	+	24	88.5					
	E14=113	33	280504	+	25	96.3					
		20	274364	+	20	100					
	E15=76	24	165239	+	22	100					
	E16=134	41	239813	+	27	93.6					
		29	298103	+	25	75					
	E17=126	20	288540	-	20	100					
	E18=211	26	302008	+	25	88.5					
		38	308741	-	24	96.3					
	E19=596	52	309222	-	49	84.7					
		54	312080	+	49	83.4					
**Elephant**	E1=371	502	20	+	29	96.8					
		30	11	+	24	93.2					
	E2=162	27	31	-	24	77.8	1-162	scaffold_6	−	35.68	0.0001172
		20	10	+	20	100	1-162	scaffold_5	−	23.28	0.2091
	E3=328	38	35	-	29	78.2	1-328	scaffold_6	−	62.46	3.56E-13
		33	6	-	25	77.8	84-263	scaffold_5	+	41.98	1.914E-07

	E4=135	100	3	+	42	93.8	1-135	scaffold_6	-	40.13	0.000198
		51	17	+	41	76.6	1-135	scaffold_5	-	27.45	0.1869
	E5=150	25	75	-	22	87.5	1-150	scaffold_6	-	54.71	3.12E-07
							1-150	scaffold_6	-	26.87	0.52
	E6=123	42	6	-	36	92.9	1-123	scaffold_6	-	71.77	3.90E-15
		27	2	+	22	100	1-123	scaffold_6	-	24.17	0.07
	E7=136	20	78	+	20	100					
	E8=700	306	6	-	147	78.3	65-395	scaffold_6	-	123.13	4.35E-33
		43	7	-	37	93.1	202-395	scaffold_6	-	57.24	4.60E-13
	E9=74										
	E10=75	21	5	-	21	100					
		22	53	-	20	95.5					
	E11=96										
	E12=119	20	34	+	20	100					
	E13=143	77	59	-	24	100					
		24	44	-	23	79.2					
	E14=113	300	60	-	32	94.6					
		21	51	+	21	100					
	E15=76										
	E16=134	23	2	-	23	100					
	E17=126	25	32	-	22	87.5					
		20	5	-	20	100					
	E18=211	111	6	-	72	80.7	1-211	scaffold_6	-	65.15	2.41E-13
		35	9	+	23	100	1-211	scaffold_5	-	21.04	0.3947
	E19=596	368	6	-	167	80.9	4-241	scaffold_6	-	105.07	1.39E-29
		161	88	+	64	93.3	355-553	scaffold_6	-	102.87	7.15E-29
**Dog**	E1=371	38	21	-	23	96	28-356	11	+	142.66	4.01E-42
		23	14	+	22	87					
	E2=162	27	9	+	24	77.8					
		25	11	+	22	100					
	E3=328	93	11	+	54	87.9	1-328	11	+	64.84	3.05E-14
		67	15	-	24	100	75-307	11	+	25.64	0.002835
	E4=135	52	28	-	28	96.8	1-135	11	+	49.73	4.44E-07
		29	35	+	25	93.2	1-135	11	+	22.65	0.9938

	E5=150	450	10	-	30	100					
		34	11	-	27	58.7					
	E6=123	71	11	+	35	86.1	1-123	11	+	35.09	2.63E-05
		23	17	+	21	95.7	1-123	11	+	21.08	0.213
	E7=136	23	18	+	23	100					
		22	36	+	22	100					
	E8=700	76	12	-	44	87.8	1-700	11	+	88.45	5.99E-23
		57	27	-	38	87	205-395	11	+	28.96	0.000072
	E9=74										
	E10=75										
	E11=96	58	28	+	27	100	1-96	11	+	44.8	8.09E-06
		282	16	+	24	100					
	E12=119	35	1	-	25	85.8					
	E13=143	42417	28	+	32	97.1					
		23	22	+	22	87					
	E14=113	62	x	-	25	100					
		23	8	+	22	87					
	E15=76	55	11	+	35	81.9	1-76	11	+	41.65	8.57E-07
							1-76	11	+	24.54	0.1093
	E16=134	21	8	+	21	100					
	E17=126	26	29	-	24	96.2	4-122	11	+	35.71	0.0003089
		20	12	-	20	100	1-126	11	−	23.99	0.3421
	E18=211	31	4	+	26	93.2	1-211	11	+	74.6	2.31E-16
		24	18	-	22	95.9	1-211	11	−	18.18	0.9774
	E19=596	58	1	-	52	81.1	308-587	11	+	130.6	3.37E-38
		58	2	-	50	86.8	248-589	11	+	40.16	4.312E-09
**Horse**	E1=371	305	23	+	164	77.9	1-371	23	+	192.62	2.36E-58
		45	10	-	33	91.9					
	E2=162	24	5	+	23	83.4	1-162	23	+	50.76	3.75E-09
	E3=328	346	23	+	60	86.6	1-328	23	+	38.04	6.92E-07
		36	9	-	28	93.8	93-254	23	−	33.47	0.000013
	E4=135	31	6	-	28	77.5	1-135	23	+	67.92	1.72E-11
		43	24	+	28	96.7	1-135	23	−	23.97	0.3507
	E5=150	32	7	-	26	82.8	1-150	23	+	74.92	2.70E-12
		30	14	-	24	100	1-150	23	+	24.77	0.4435

	E6=123	73	20	-	28	96.7	1-123	23	+	63.92	1.73E-13
		20	21	-	20	100	1-123	23	+	21.45	0.121
	E7=136	22	4	+	20	95.5					
	E8=700	45	1	+	37	91.2	1-700	23	+	139.33	1.49E-38
		44	11	+	36	91	202-395	23	+	43.59	1.86E-09
	E9=74	26	21	-	24	100					
		20	9	-	20	100					
	E10=75										
	E11=96	404	29	-	30	94.2					
	E12=119	40	1	-	29	96.9					
		20	29	+	20	100					
	E13=143	45	3	+	23	96	1-143	23	+	104.78	8.02E-23
		20	U	-	20	100	1-143	23	+	34.51	0.000088
	E14=113	21	11	+	21	100	1-113	23	+	40.48	6.09E-05
		20	6	+	20	100	1-113	23	+	26.46	0.1377
	E15=76						1-76	23	+	58.48	5.83E-12
							1-76	23	-	23.63	0.148
	E16=134	78	23	+	56	85.9	1-134	23	+	58.86	1.91E-11
		27	20	+	24	73.1	1-134	23	-	21.12	0.2329
	E17=126	25	1	-	22	100	19-109	23	+	34.97	0.0003477
		20	10	-	20	100	1-126	23	-	26.64	0.05058
	E18=211	23	10	-	21	95.7	1-211	23	+	96.3	1.63E-22
		20	26	-	20	100	1-211	23	-	17.65	0.9864
	E19=596	198	23	+	125	83	294-587	23	+	132.58	5.59E-39
		191	12	-	62	94.4	245-592	23	+	33.22	0.000001
**Cow**	E1=371	36	11	+	27	96.7	1-371	8	-	194.34	1.39E-58
		27	9	+	26	85.2					
	E2=162						1-162*	8	+	27.88	0.007844
							1-162	8	-	26.34	0.01982
	E3=328	78	15	-	69	77	1-328	8	-	48.98	1.27E-09
		71	13	-	54	72.6	78-269	8	+	35.38	0.000008
	E4=135	152	8	-	55	91.2	1-135	8	-	69.56	1.49E-11
		39	6	-	33	71.1	1-135	8	-	25.52	0.3192
	E5=150	114	8	-	81	80.8	1-150	8	-	67.49	2.60E-10
		57596	U_JH122057	+	26	96.6	1-150	8	-	25.82	0.5415
	E6=123	40	14	+	23	100	1-123	8	-	66.06	9.18E-14
							1-123	8	+	26.23	0.01176

	E7=136	1134	U_AAFC03097047	+	67	90.6					
		826	U_AAFC03091024	+	64	89.1					
	E8=700	443	14	-	40	97.8	1-700	8	−	71. 74	1. 08E-17
		326892	11	+	38	97.5	201-395	8	+	36. 14	0. 000001
	E9=74	515	15	-	24	100					
	E10=75	183	12	-	28	100					
		28	5	-	22	100					
	E11=96	435	20	+	25	100					
		20	28	+	20	100					
	E12=119	1490	U_AAFC03097055	-	50	90.5					
		259	10	+	49	93					
	E13=143	32	2	-	23	96.2	1-143	8	−	83. 19	5. 89E-17
		279297	U_JH121298	-	22	92.4	1-143	8	+	33. 73	0. 000293
	E14=113	53	8	-	45	92.5	1-113	8	−	65. 28	1. 48E-10
		372	19	-	29	94					
	E15=76	40	8	-	32	88.9	1-76	8	−	29. 72	0. 004704
		26	7	+	20	88.5	1-76	8	+	23. 13	0. 4361
	E16=134	69	12	+	25	100					
		20	6	+	20	100					
	E17=126	20	2	-	20	100					
	E18=211	27	5	+	24	65.4	1-211	8	−	66. 43	6. 42E-14
		22	15	-	22	100	1-211	8	+	19. 17	0. 7849
	E19=596	156	8	-	74	83.4	281-587	8	−	96. 11	6. 42E-27
		69	x	+	55	83.9	246-591	8	−	23. 99	0. 00104
**Rabbit**	E1=371	275	1	-	97	92.2	1-371	1	−	136. 85	7. 482E-40
		124	x	-	30	94.2					
	E2=162	36	2	-	24	96.2					
		32	3	-	23	96					
	E3=328	309	7	-	28	100	1-328	1	−	28	0. 001565
		25	1	+	24	80	81-266	1	+	29. 85	0. 0004752
	E4=135	32	15	-	30	78.2	1-135	1	−	71. 63	8. 138E-12
		32	7	+	27	82.8	1-135	1	+	26. 34	0. 3419
	E5=150	208	4	-	24	100	1-150	1	−	91. 88	1. 514E-15
		39	2	-	23	100	1-150	1	−	27. 5	0. 3813
	E6=123										
	E7=136										

	E8=700	301	1	-	127	77.7	1-700	1	-	162.54	4.591E-45
		36	4	+	31	85.8	202-395	1	+	24	0.005807
	E9=74	26	13	-	23	100					
		30	16	+	22	100					
	E10=75	71	9	+	25	100					
		27	9	+	25	100					
	E11=96	24	4	-	22	95.9					
	E12=119	270	2	-	33	100					
		431	U0011	+	27	96.7					
	E13=143	36	7	-	25	96.3	1-143	1	-	68.05	7.556E-13
		25	16	+	23	96	1-143	1	+	28.86	0.008709
	E14=113	23	4	+	21	100	1-113	1	-	34.09	0.007216
		20	11	+	20	100					
	E15=76						1-76	1	-	28.48	0.01839
	E16=134	25	2	+	24	76					
		79	12	+	24	100					
	E17=126	23	17	+	21	95.7	1-126	1	-	50.77	9.579E-08
		20	17	-	20	100	1-126	1	+	36.09	0.0006194
	E18=211	31	U0265	+	24	100	1-211	1	-	73.7	1.033E-15
		38	1	-	23	100	1-211	1	-	21.93	0.2251
	E19=596	168	1	-	83	88.2	320-587#	1	-	99.67	7.629E-28
		121	1	-	53	79.4	4-241#	1	-	68.85	6.332E-18
**Guinea Pig**	E1=371	333754	14	+	39	100	1-371	scaffold_2	+	76.24	1.94E-20
		25	22	+	25	100					
	E2=162	28	8	+	22	100					
	E3=328	75	12	+	33	59.5	1-328	scaffold_2	+	30.43	0.000258
		51	27	+	31	80.4	81-266	scaffold_2	-	24.1	0.01519
	E4=135	39	24	-	26	93.4	1-135	scaffold_2	+	80.57	0.000258
		260	168	-	26	96.6	1-135	scaffold_2	+	24.61	0.6848
	E5=150										
	E6=123	37	21	-	27	65.6	1-123	scaffold_2	+	25.92	0.01899
		22	55	-	22	100	1-123	scaffold_2	+	24.35	0.05199
	E7=136	107	15	-	68	87.5					
		84	14	+	57	81.5					
	E8=700	53	4	+	36	78.8	1-700	scaffold_2	+	81.68	1.36E-20
		39	5	-	31	89.8	202-395	scaffold_2	+	20.25	0.06358

	E9=74	391	31	+	25	100					
	E10=75	22	8	-	22	100					
	E11=96	21	11	+	21	100					
	E12=119	256	2	-	47	91.3					
		127	66	+	47	94.5					
	E13=143						1-143	scaffold_2	+	50.76	1.64E-08
							1-143	scaffold_2	1	26.12	0.03476
	E14=113	33	30	-	24	96.2					
		31	54	+	24	100					
	E15=76	20	283	-	20	100	1-76	scaffold_2	+	36.37	6.40E-05
							1-76	scaffold_2	1	24.56	0.2146
	E16=134	76	2	+	50	82.9	1-134	scaffold_2	+	53.01	1.92E-09
		23	26	-	23	100	1-134	scaffold_2	1	21.67	0.4588
	E17=126										
	E18=211	299	15	-	34	92.4					
		22	44	-	20	95.5					
	E19=596	92	156	-	61	98.6	310-587#	scaffold_2	+	75.2	4.55E-20
		67	3	-	54	96.7	13-129#	scaffold_2	+	21.29	0.01014
**Kangaroo Rat**	E1=371	33	5694	-	25	96.5	28-180	GeneScaffold_1017	+	31.18	6.10E-06
		28	0	-	25	64.3					
	E2=162	26	167	+	23	77					
		20	6198	-	20	100					
	E3=328	21	19945	+	21	100	328	GeneScaffold_1017	+	29.65	0.000415
		20	1642	+	20	100	81-268	GeneScaffold_5830	1	44.55	2.81E-08
	E4=135	31	6711	+	26	67.9	135	GeneScaffold_1017	+	33.37	0.005903
		24	132020	-	23	79.2	135	GeneScaffold_1445	-	27.32	0.1549
	E5=150	150	210	+	118	89.4	150	GeneScaffold_1017	+	127.94	1.01E-23
		35	36492	+	24	92.9					
	E6=123	24	1921	+	22	100	123	GeneScaffold_1017	+	41.93	6.31E-07
		20	46256	-	20	100	123	GeneScaffold_6837	-	25.19	0.0161
	E7=136	72	856	+	41	78.8					
		526	13685	+	39	95.5					
	E8=700	35	298	-	23	100					
		24	1562	+	22	100					
	E9=74	26	35416	-	23	100					
		23	4051	-	21	100					

	E10=75										
	E11=96	21	2622	+	21	100					
		21	2622	+	21	100					
	E12=119	94	3960	+	40	91.7					
		35	24954	-	30	97					
	E13=143										
	E14=113										
	E15=76										
	E16=134	20	42896	-	20	100					
		20	15144	-	20	100					
	E17=126	23	5154	-	21	100					
		20	36887	-	20	100					
	E18=211	41	12528	+	25	96.3					
		25	12734	+	21	95.9					
	E19=596	21577	1	-	70	93.9					
		58	79	+	54	82.8					
**Rat**	E1=371	33	5	-	25	100					
		25	7	+	24	88					
	E2=162	30	7	+	26	79.4					
		300	15	+	25	100					
	E3=328	30	8	-	28	70					
		378	7	+	26	100					
	E4=135	124	5	-	30	96.9					
		29	14	-	27	72.5					
	E5=150	472	2	+	30	100					
		22	9	-	20	95.5					
	E6=123	67	18	+	24	100					
	E7=136	203891	1	-	50	90.2					
		78	12	+	44	77.2					
	E8=700	26	3	-	25	88.5					
		26	2	-	22	92.4					
	E9=74	20	7	-	20	100					
	E10=75	23	1	-	22	87					

	E11=96	22	9	-	22	100					
	E12=119	81	6	+	33	94.6					
		66	2	+	30	96.9					
	E13=143	30	8	+	24	90					
		22	5	-	20	95.5					
	E14=113	21	5	+	21	100					
	E15=76										
	E16=134	20	8	-	20	100					
		20	20	-	20	100					
	E17=126	20	15	-	20	100					
	E18=211	38	11	+	24	96.3					
		20	14	-	20	100					
	E19=596	303837	1_random	-	60	97.1					
		87	4	-	57	92.7					
**Mouse**	E1=371	28	1	-	25	66.7					
		32	10	+	25	96.3					
	E2=162	34	4	-	24	100					
		22	5	+	20	95.5					
	E3=328	100	19	+	30	100					
		46	6	+	28	93.6					
	E4=135	39	6	-	27	966					
		21	13	+	21	100					
	E5=150	36	13	+	29	74.2					
		32	17	+	25	70.4					
	E6=123										
	E7=136	162484	1	+	55	93.6					
		373547	1	+	47	94.4					
	E8=700	24	1	-	24	100					
		22	16	-	22	100					
	E9=74										
	E10=75										
	E11=96										

	E12=119	72	12	-	33	86.2					
		94	6	+	33	67.5					
	E13=143	23	X	+	22	87					
		20	1	-	20	100					
	E14=113	22	17	-	20	95.5					
		20	19	+	20	100					
	E15=76										
	E16=134	26	14	-	24	100					
		30	11	+	24	90					
	E17=126	20	9	-	20	100					
		20	2	+	20	100					
	E18=211	31	9	-	25	90.4					
		27	4	+	24	69.3					
	E19=596	236	1	-	58	95.4					
		84	7	-	57	93.8					
**Tree Shrew**	E1=371	29	11608.1-153621	-	26	82.8					
		24	12793.1-295593	-	24	100					
	E2=162	26	137324.1-225074	-	23	84	1-162	GeneScaffold_4495	-	24.16	0.09483
	E3=328	46	149005.1-175128	-	41	64.5	1-328	GeneScaffold_4495	-	42.87	8.299E-08
		45	113065.1-313317	-	35	88.9	1-328	GeneScaffold_4495	-	42.87	8.299E-08
	E4=135	26	121089.1-179439	+	25	80.8	1-135	GeneScaffold_4495	-	55.12	4.687E-08
		24	117713.1-193458	-	23	79.2	1-135	scaffold_143574	-	27.99	0.1079
	E5=150	20	138816.1-165090	-	20	100					
		20	123001.1-225613	-	20	100					
	E6=123	21	150588.1-190788	+	21	100					
	E7=136	55	14353.1-406122	-	44	85.2					
		72	129769.1-51628	+	42	72					
	E8=700	62	150588.1-190788	-	46	87.1					
		37	131051.1-185533	+	34	89.2					
	E9=74	167	134928.1-13767	-	27	96.6					
		30	130710.1-187393	+	22	95.9					
	E10=75	71	103637.1-14656	-	49	86.6					
		32	115074.1-137271	-	28	96.8					
	E11=96						1-96	GeneScaffold_4495	-	47.55	0.000003499
	E12=119	91	143312.1-125063	+	49	75.9					
		186	93744.1-11733	+	38	89.6					

	E13=143	21	100804.1-9163	+	21	100	1-143	GeneScaffold_4495	-	39.14	0.00001541
							1-143	scaffold_134426	-	23.87	0.1279
	E14=113	28	118084.1-760553	-	25	74.1	1-113	GeneScaffold_4495	-	44.19	0.00002124
		33	134874.1-270347	+	25	100					
	E15=76										
	E16=134	22	124158.1-7356	-	22	100					
	E17=126	27	8446.1-217465	-	24	96.2					
		24	129914.1-265838	+	22	95.9					
	E18=211	38	117713.1-193458	+	35	75.7	1-211	GeneScaffold_4495	-	68.75	1.877E-14
		24	150503.1-305525	-	23	79.2	1-211	scaffold_113228	-	22.12	0.1531
	E19=596	268	11122.1-59405	+	60	94.1					
		54	126320.1-397028	-	46	92.6					
**Mouse Lemur**	E1=371	178	1070	+	105	84	181-329	scaffold_1070	+	22.01	0.005474
		311	2881	-	32	97.2					
	E2=162	27	5065	+	24	77.8	1-162	scaffold_1070	+	60.39	3.029E-11
		25	1358	-	23	100	1-162	GeneScaffold_1621	-	33.39	0.000361
	E3=328	74	958	-	61	73	1-328	scaffold_1070	+	58	4.852E-12
		82	15402	+	57	74.3	80-268	GeneScaffold_299	-	29.89	0.000355
	E4=135	123	1070	+	81	82.2	1-135	scaffold_1070	+	90.94	1.854E-16
		32	767	-	27	87.1	1-135	scaffold_1591	+	27.2	0.165
	E5=150	128	1070	+	98	88.9	1-150	scaffold_1070	+	121.47	2.815E-22
		142	10817	-	27	93.6					
	E6=123	123	1070	+	83	83.8	1-123	scaffold_1070	+	93.98	1.921E-21
		20	116145	-	20	100	1-123	GeneScaffold_2064	-	21.47	0.3225
	E7=136	117	3233	+	69	88	1-136	scaffold_1070	+	72.69	2.27E-14
		107	30	-	68	80	1-136	GeneScaffold_2308	+	60.6	3.71E-11
	E8=700	159299	1144	+	42	95.8					
		403	847	+	34	100					
	E9=74	444	80721	+	27	93.6					
		33	6243	+	26	70					
	E10=75	64	432	+	46	87.4					
		28	2502	+	27	89.3					
	E11=96	49	1070	+	42	93.8	1-96	scaffold_1070	+	73.83	2.074E-12
							1-96	GeneScaffold_2001	+	27.1	0.245
	E12=119	135	79	+	87	90.8	1-119	scaffold_1070	+	49.62	2.16E-08
		136	91	+	85	89.1	1-119	GeneScaffold_3375	-	88.57	1.04E-18
	E13=143	104	11002	-	27	96.6					
		23	901	-	21	95.7					

	E14=113	98	1070	+	62	87.1	1-113	scaffold_1070	+	48.03	0.000002552
		23	132	+	22	87					
	E15=76	28	3288	-	22	95.9					
	E16=134	89	638	+	24	100	1-134	scaffold_1070	+	42.47	0.000001228
							1-134	GeneScaffold_3279	+	24.52	0.07728
	E17=126	23	834	-	21	95.7					
		21	30	-	21	100					
	E18=211	46	9700	+	24	96.3					
		24	133193	-	22	100					
	E19=596	120	355	+	50	76					
		53	51	+	49	81.2					
**Tarsier**	E1=371	43	3144	+	25	96.3					
		29	33897	-	24	77.8					
	E2=162	27	10137	-	26	74.1	1-162	scaffold_3388	+	31.09	0.001449
		27	440905	-	23	88	1-162	GeneScaffold_5108	-	32.01	0.000828
	E3=328	86	3388	+	74	87.1	1-328	scaffold_3388	+	84.78	1.557E-19
		83	525961	-	66	73.5	83-264	scaffold_3641	+	42.55	1.019E-07
	E4=135	67	248383	+	29	96.8					
		27	411220	-	26	70.4					
	E5=150	111	3388	+	82	88.1	1-150	scaffold_3388	+	106.96	4.94E-19
		27	42816	-	25	77.8					
	E6=123	20	15092	-	20	100	1-123	scaffold_3388	+	36.02	0.00002808
							1-123	scaffold_7921	-	24.33	0.05104
	E7=136	117	5427	+	73	92	1-136	scaffold_3388	+	52.78	0.05195
		108	3407	+	67	86.1	1-136	scaffold_3388	-	66.13	1.258E-12
	E8=700	88	1412	+	45	91					
		62	8886	+	42	83.9					
	E9=74	35	44235	+	23	100					
		23	39658	+	22	87					
	E10=75	69	3534	+	52	90.7					
		114	137510	+	28	96.9					
	E11=96	54	8886	+	48	94.5					
		23	452194	+	21	100					
	E12=119	141	17473	-	75	92.2					
		137	38709	+	73	90.2					
	E13=143	27	19476	-	24	100					
		26	151605	+	24	96.2					
	E14=113	33	6797	+	23	96					
		20	5216	+	20	100					

	E15=76						1-76	scaffold_13816	+	43.5	4.642E-07
							1-76	scaffold_40813	-	25.4	0.1171
	E16=134	62	13816	+	52	92	1-134	scaffold_13816	+	66.23	5.478E-13
							1-134	scaffold_4635	-	22.14	0.3341
	E17=126	327	482722	-	24	100	1-126	scaffold_13816	+	81.8	6.481E-16
		326	15314	+	24	100	1-126	scaffold_14481	-	29.07	0.03162
	E18=211	69	13816	+	49	85.6	1-211	scaffold_13816	+	97.79	1.69E-22
		24	44096	-	23	87.5	1-211	scaffold_13473	+	19.95	0.6108
	E19=596	437	13816	+	188	85.4	296-587	scaffold_13816	+	134.36	4.026E-39
		60	3416	+	54	81.7	248-589	scaffold_9615	+	27.31	0.000114
**Marmoset**	E1=371	364	1	+	311	91	1-371	1	+	421.17	2.31E-131
		47	4	+	28	100					
	E2=162	162	1	+	135	92.5	1-162	1	+	141.27	2.686E-32
		26	8	-	23	76	1-162	1	+	22.39	0.3878
	E3=328	319	1	+	241	88	1-328	1	+	259.07	3.738E-68
		179	1	-	120	90	78-269	1	-	86.09	9.37E-20
	E4=135	135	1	+	107	91.5	1-135	1	+	144.19	8.451E-29
		316017	16	+	42	97.9	1-135	1	+	29.42	0.06968
	E5=150	146	1	+	132	95.3	1-150	1	+	167.07	2.5E-32
		494	3	+	31	94.2	1-150	1	-	31.72	0.04668
	E6=123	127	1	+	100	91.1	1-123	1	+	126.52	2.251E-30
		3518	Jn_ACFV0119136	-	20	100	1-123	1	+	30.91	0.00105
	E7=136	115	12	-	97	92.2	1-136*	1	-	126.26	1.84E-28
		117	16	-	96	92.2	1-136	1	+	85.07	1.632E-17
	E8=700	699	1	+	539	90.4	1-700	1	+	637.27	2.661E-189
		316	X	-	233	90.4	1-700	1	-	239.7	1.76E-68
	E9=74	39	8	+	24	96.2	1-74	1	+	71.89	2.804E-11
		20	6	+	20	100					
	E10=75	64	1	+	58	95.4	1-75	1	+	86.85	1.8E-20
		70	7	-	54	89.8	1-75	1	-	29.89	0.004163
	E11=96	95	1	+	77	90.6	1-96	1	+	93.29	7.135E-17
							1-96	1	+	28.62	0.1496
	E12=119	119	1	+	93	89.1	1-119	1	+	118.67	1.535E-26
		138	18	-	88	88.7	1-119	1	-	92.05	1.74E-19
	E13=143	139	1	+	114	91.4	1-143	1	+	161.42	8.738E-37
		39	8	-	26	100	1-143	1	-	33.53	0.0005926
	E14=113	111	1	+	88	92.4	1-113	1	+	110.85	3.311E-21
		231	4	-	32	94.3	1-113	1	+	25.33	0.973
	E15=76	22	15	-	22	100	1-76	1	+	81.07	3.955E-18
							1-76	1	-	27.51	0.03878

	E16=134	85	1	+	70	88.3	1-134	1	+	91.12	1.716E-19
		20	3	-	20	100	1-134	1	+	23.31	0.2286
	E17=126	21	8	+	21	100	1-126	1	+	100.68	1.147E-20
		20	10	+	20	100	1-126	1	+	29.5	0.03448
	E18=211	210	1	+	170	90.5	1-211	1	+	210.71	1.277E-53
		20	15	+	20	100	1-211	1	-	21.49	0.3206
	E19=596	224	1	+	183	90.4	10-228#	1	+	157.43	2.12E-46
		87	1	+	80	93.2	249-588#	1	+	32.53	3.34E-06
**Macaque**	E1=371	371	15	-	333	94.9	1-371	15	-	484.38	5.364E-152
		351	11	+	34	94.6					
	E2=162	160	15	-	148	96.3	1-162	15	-	194.89	1.237E-46
		86	15	-	70	90.7	1-162	15	+	26.74	0.0147
	E3=328	311	15	-	284	94.6	1-328	15	-	132.68	4.517E-33
		179	15	+	134	92.2	78-269	15	+	24.48	0.008515
	E4=135	135	15	-	118	94.8	1-135	15	-	173.09	7.389E-36
		35	X	+	30	96.9	1-135	15	-	30.94	0.01604
	E5=150	147	15	-	134	92.6	1-150	15	-	173.61	4.498E-34
		28	17	-	23	96					
	E6=123	122	15	-	109	92.7	1-123	15	-	140.55	1.444E-34
		39	2	-	25	100	1-123	15	-	23.74	0.05511
	E7=136	124	12	+	97	84.3	1-136	15	-	170.45	1.731E-40
		117	2	-	96	92.2	1-136	15	-	136.58	1.744E-31
	E8=700	699	15	-	619	95.4	1-700	15	-	846.72	3.107E-253
		317	X	+	240	89.3	1-700	15	-	252.03	1.65E-72
	E9=74	74	15	-	62	91.9	1-74	15	-	89.62	1.052E-15
		25	5	-	24	84					
	E10=75	75	15	-	69	96	1-75	15	-	101.89	2.457E-25
		425	1	+	57	89.2	1-75	15	+	22.27	0.4598
	E11=96	96	15	-	92	98	1-96	15	-	117.5	6.873E-23
		20	12	-	20	100					
	E12=119	107	15	-	95	94.4	1-119	15	-	149.28	6.239E-35
		143	4	+	86	90.7	1-119	15	-	90.5	2.345E-19
	E13=143	143	15	-	131	95.9	1-143	15	-	191.11	1.092E-44
		34	7	+	29	96.9	1-143	15	+	27.78	0.009274
	E14=113	113	15	-	95	95.3	1-113	15	-	129.37	6.365E-26
		268	4	+	26	96.5					
	E15=76	76	15	-	66	93.5	1-76	15	-	105.7	9.18E-26
							1-76	15	+	25.42	0.08514
	E16=134	133	15	-	122	94	1-134	15	-	160.14	3.216E-38
		184	1	+	27	96.6	1-134	15	-	22.23	0.232

	E17=126	106	15	-	97	92.5	1-126	15	-	146.6	7.217E-33
		20	7	+	20	100	1-126	15	-	25.17	0.2404
	E18=211	211	15	-	185	93.9	1-211	15	-	263.39	1.714E-68
		24	11	-	23	87.5	1-211	15	-	18.71	0.9882
	E19=596	539	15	-	478	92.5	1-596	15	-	635.42	1.63E-200
		142	X	-	88	85.5	250-587	15	+	39.18	1.27E-08
**Orangutan**	E1=371	371	9	-	359	98.4	1-371	9	-	482.43	2.79E-151
		27	15	+	25	100					
	E2=162	157	9	-	141	96.1	1-162	9	-	154.2	7.048E-36
		60	9	-	52	93.4	1-162	9	+	20.32	0.8676
	E3=328	330	9	-	300	98.1	1-328	9	-	307.29	7.734E-82
		198	9	+	146	94.6	81-266	9	+	98.53	1.99E-23
	E4=135	133	9	-	123	96.3	1-135	9	-	165.16	6.561E-34
		57	14	+	29	87.9	1-135	9	+	25.38	0.3971
	E5=150	150	9	-	140	96.7	1-150	9	-	180.35	1.717E-35
		38	8	-	28	83.9	1-150	9	+	27.55	0.2579
	E6=123	123	9	-	113	96	1-123	9	-	146.58	3.695E-36
		21	X	-	21	100	1-123	9	-	24.13	0.05255
	E7=136	136	9	-	118	93.4	1-136	9	-	149.03	1.051E-34
		137	26	+	102	91.2	1-136	9	+	127.79	4.649E-29
	E8=700	699	9	-	653	97.2	1-700	9	-	816.99	4.132E-244
		315	7	+	241	90.4	1-700	9	-	252.38	1.593E-72
	E9=74	34	11_random	+	24	100					
		21	4	-	21	100					
	E10=75	416	1	+	44	91					
		52	1	-	40	90					
	E11=96	96	9	-	92	98	1-96	9	-	118.16	5.893-23
		25	12	+	24	76					
	E12=119	119	9	-	111	96.7	1-119	9	-	147.88	1.805E-34
		120	15_random	-	88	90.7	1-119	9	-	92.85	6.86E-20
	E13=143	143	9	-	135	97.3	1-143	9	-	188.51	6.228E-44
		24	3	+	22	95.9	1-143	9	-	33.89	0.0003091
	E14=113	107	9	-	101	97.2	1-113	9	-	125.72	5.862E-25
		24	7	+	23	87.5					
	E15=76	76	9	-	72	97.4	1-76	9	-	100.43	4.218E-24
							1-76	9	-	25.4	0.1058
	E16=134	134	9	-	126	97.1	1-134	9	-	151.03	1.076E-35
		24	3	+	22	95.9	1-134	9	-	22.08	0.3117
	E17=126	119	9	-	115	98.4	1-126	9	-	146.31	1.056E-32
		21	4	-	21	100	1-126	9	+	27.87	0.05851

	E18=211	211	9	-	205	98.6	1-211	9	-	268.91	6.256E-70
		25	6	+	24	84	1-211	9	-	21.33	0.229
	E19=596	603	9	-	557	97.3	1-596	9	-	674.37	5.832E-213
		173	20	-	59	95.6	247-590	9	+	32.29	0.000002573
**Gorilla**	E1=371	371	9	+	365	99.2	1-371	9	+	493.1	9.311E-155
		23	15	+	21	95.7					
	E2=162	162	9	+	160	99.4	1-162	9	+	196.8	4.351E-47
		86	9	+	70	90.7	1-162	9	+	196.8	0.3084
	E3=328	312	9	+	301	97.5	1-328	9	+	338.47	1.32E-90
		180	9	-	130	93.2	81-266	9	-	107.89	4.33E-26
	E4=135	133	9	+	131	99.3	1-135	9	+	172.3	1.257E-35
		62216	11	-	34	97.3	1-135	9	+	24.25	0.6616
	E5=150	150	9	+	148	99.4	1-150	9	+	185.72	9.799E-37
		46	6	-	26	100	1-150	9	-	26.88	0.3267
	E6=123	123	9	+	123	100	1-123	9	+	156.33	6.367E-39
		25	12	+	22	87.5	1-123	9	-	22.09	0.1759
	E7=136	136	9	+	124	95.6	1-136	9	+	155.81	1.498E-36
		131	15	+	101	88.6	1-136	9	+	133.58	1.217E-30
	E8=700	695	9	+	676	98.5	1-700	9	+	827.23	2.887E-247
		317	x	-	240	92	1-700	9	+	251.88	2.041E-72
	E9=74	74	9	+	72	98.7	1-74	9	+	89.91	9.968E-16
		374	7	-	28	100					
	E10=75	75	9	+	71	97.4	1-75	9	+	96.56	1.155E-23
		24	6	+	22	95.9	1-75	9	-	23.79	0.1758
	E11=96	96	9	+	96	100	1-96	9	+	122.7	4.473E-24
							1-96	9	+	31.61	0.01707
	E12=119	119	9	+	109	95.8	1-119	9	+	147.13	2.567E-34
		132	14	-	85	90.4	1-119	9	-	52.29	3.47E-09
	E13=143	143	9	+	143	100	1-143	9	+	193.21	3.518E-45
		27	8	+	23	92.6	1-143	9	+	31.94	0.000881
	E14=113	107	9	+	105	99.1	1-113	9	+	128.26	1.305E-25
	E15=76	76	9	+	76	100	1-76	9	+	104.96	1.692E-25
							1-76	9	+	25.4	0.0957
	E16=134	134	9	+	132	99.3	1-134	9	+	163.76	3.841E-39
		23	9	-	21	100	1-134	9	+	20.51	0.7416
	E17=126	123	9	+	121	99.2	1-126	9	+	154.18	8.637E-35
		38	6	-	28	71.9	1-126	9	+	27.95	0.05045
	E18=211	211	9	+	201	97.7	1-211	9	+	256.02	2.092E-66
		20	9	-	20	100					

	E19=596	595	9	+	563	97.8	1-596	9	+	685.11	1.843E-216
		490	8	-	77	93.4	247-590	9	-	41.61	2.333E-09
**Chimpanzee**	E1=371	371	9	+	367	99.5	1-371	9	+	486	2.029E-152
		1892628	_GL391837_rando	+	28	100					
	E2=162	162	9	+	156	98.2	1-162	9	+	190.85	1.787E-45
		86	9	+	68	89.6	1-162	9	+	20.25	0.9289
	E3=328	313	9	+	300	98.4	1-328	9	+	347.96	3.3E-93
		181	9	-	133	95.3	81-266	9	-	119.07	3.66E-29
	E4=135	133	9	+	129	98.5	1-135	9	+	169.76	5.6E-35
		363842	17	-	41	95.5					
	E5=150	150	9	+	146	98.7	1-150	9	+	183.54	3.411E-36
		46	6	-	26	100	1-150	9	-	26.23	0.5209
	E6=123	123	9	+	121	99.2	1-123	9	+	153.57	4.227E-38
		21	6	-	21	100	1-123	9	-	23.24	0.09544
	E7=136	136	9	+	128	97.1	1-136	9	+	168.75	6.177E-40
		132	2B	+	106	93.5	1-136	9	+	93.73	5.38E-20
	E8=700	696	9	+	684	99.2	1-700	9	+	864.56	1.477E-258
		326	6	+	247	91.1	1-700	9	+	200.77	7.87E-57
	E9=74	74	9	+	68	96	1-74	9	+	90.37	8.824E-16
		374	7	-	28	100					
	E10=75	75	9	+	75	100	1-75	9	+	104.25	5.916E-26
		418	1	-	54	86.7	1-75	9	+	23.14	0.3139
	E11=96	96	9	+	96	100	1-96	9	+	122.7	5.066E-24
		26	13	-	23	100	1-96	9	+	31.73	0.01802
	E12=119	119	9	+	111	96.7	1-119	9	+	147.57	2.228E-34
		142	11	-	87	91.5	1-119	9	-	97.93	3.164E-21
	E13=143	143	9	+	143	100	1-143	9	+	193.21	3.985E-45
		490	11	+	27	100	1-143	9	+	31.91	0.001018
	E14=113	107	9	+	105	99.1	1-113	9	+	128.39	1.38E-25
		20	5	-	20	100					
	E15=76	76	9	+	74	98.7	1-76	9	+	103.03	7.216E-25
							1-76	9	+	25.4	0.1084
	E16=134	134	9	+	132	99.3	1-134	9	+	163.76	4.35E-39
							1-134	9	-	22.77	0.2097
	E17=126	121	9	+	117	98.4	1-126	9	+	152.51	2.652E-34
		25	18	-	22	100	1-126	9	+	27.02	0.1
	E18=211	211	9	+	203	98.2	1-211	9	+	254.43	6.554E-66
		26	11	+	23	80					
	E19=596	600	9	+	567	98	1-596	9	+	699.06	6.795E-221
		144	1	-	66	96	260-587	9	+	36.38	1.27E-07
