# Supplementary material for: ANRIL/CDKN2B-AS shows two-stage clade-specific evolution and becomes conserved after transposon insertions in simians
Source: BMC Evol Biol. 2013 Nov 13;13:247. doi: 10.1186/1471-2148-13-247 (PMC3831594; doi:10.1186/1471-2148-13-247)
Supplement: Additional file 2 — The supplementary figures and tables. [file 1471-2148-13-247-S2.docx]

**ANRIL/CDKN2B-AS shows two-stage clade-specific evolution and becomes conserved after transposon insertions in simians**

Sha He ^1^, Weiling Gu ^1^, Yize Li ^1^, Hao Zhu ^1^**^§^**

1 Bioinformatics Section, School of Basic Medical Sciences, Southern Medical University, Shatai Road, Guangzhou, 510515, China


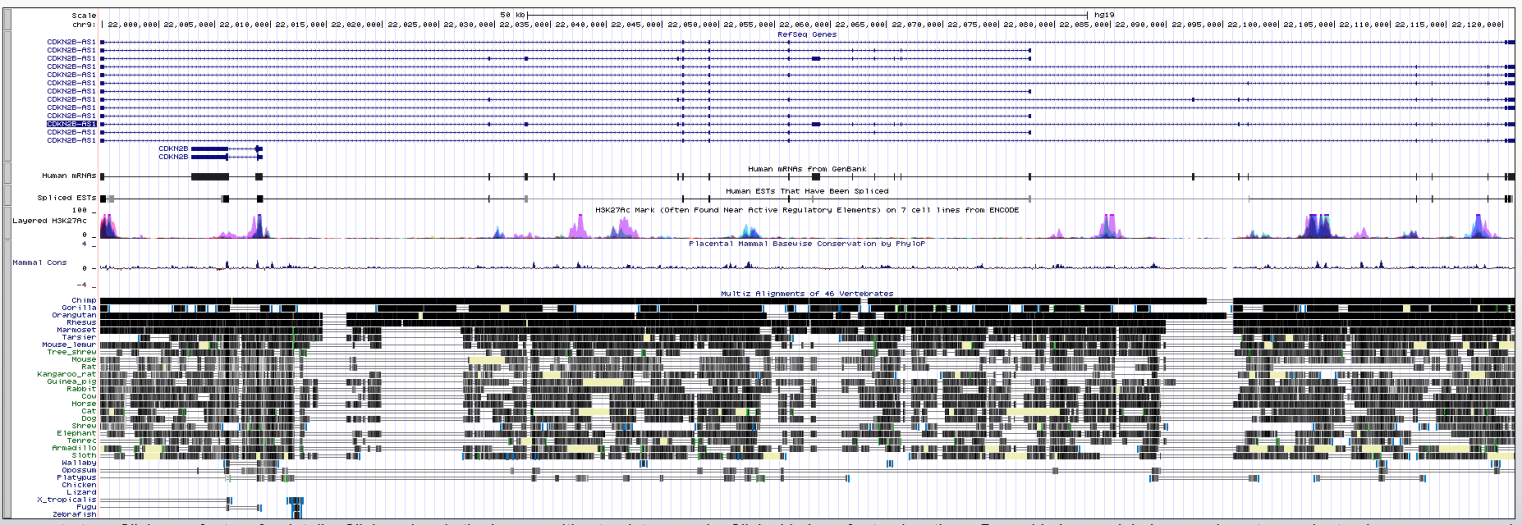


**Figure S1 The MultiZ-aligned region of the human ANRIL gene in the 27 organisms in the UCSC Genome Browser.** No aligned sequences, or extremely poorly aligned sequences, exist in vertebrates and non-placental mammals.


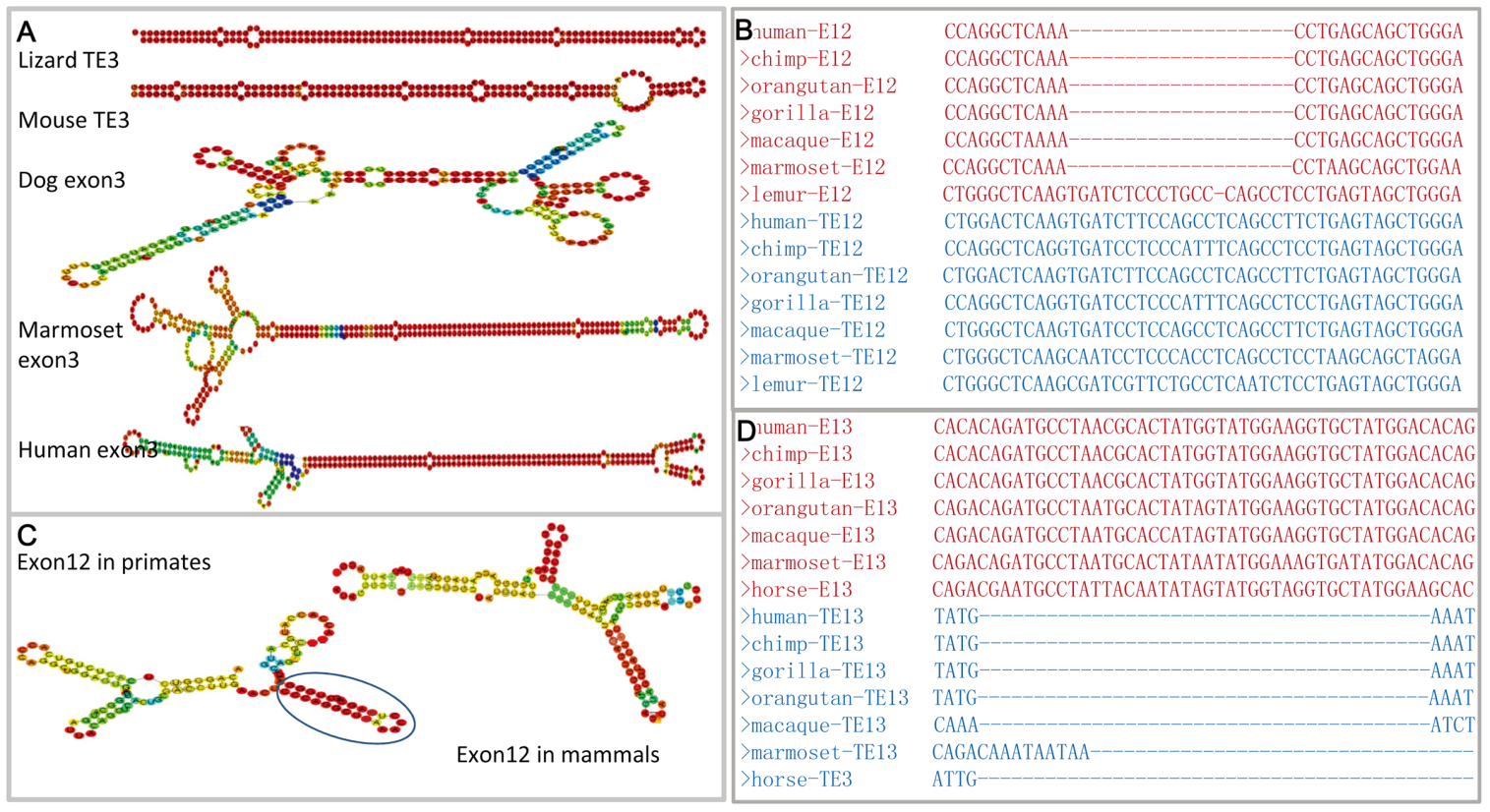


**Figure S2 Inserted transposons have modified the sequence and structure of exons.** Structures were predicted by RNAfold and RNAalifold with default parameters. (A) The palindromic structure of TE3 causes a conserved stem structure in vertebrates and mammals. The structures of simian, prosimian, and mammalian exon 3 with and without E3TE3 are quite different. (B) Compared with the highest-scoring TE12, exon 12 lost a short 20 bp sequence at a conserved position in simians. (C) Without the 20 bp sequence, a more stable hairpin structure (the marked region) forms in exon 12 in simians. (D) Compared with the highest-scoring TE13, simian (and horse) exon 13 gained a short 40 bp sequence at a conserved position.

| **Accession** | **ID** | **Query start** | **Query end** | **Subject start** | **Subject end** | **Strand** | **Score** | **Evalue** | **Alignment** |
| --- | --- | --- | --- | --- | --- | --- | --- | --- | --- |
| [MI0003660](http://www.mirbase.org/cgi-bin/mirna_entry.pl?acc=MI0003660) | [hsa-mir-645](http://www.mirbase.org/cgi-bin/mirna_entry.pl?acc=MI0003660) | 80 | 116 | 29 | 65 | + | 140 | 5e-05 | [Align](http://www.mirbase.org/cgi-bin/blast.pl#hsa-mir-645) |
| [MI0008835](http://www.mirbase.org/cgi-bin/mirna_entry.pl?acc=MI0008835) | [ptr-mir-645](http://www.mirbase.org/cgi-bin/mirna_entry.pl?acc=MI0008835) | 80 | 116 | 28 | 64 | + | 140 | 5e-05 | [Align](http://www.mirbase.org/cgi-bin/blast.pl#ptr-mir-645) |
| [MI0015121](http://www.mirbase.org/cgi-bin/mirna_entry.pl?acc=MI0015121) | [ppy-mir-645](http://www.mirbase.org/cgi-bin/mirna_entry.pl?acc=MI0015121) | 80 | 116 | 29 | 65 | + | 140 | 5e-05 | [Align](http://www.mirbase.org/cgi-bin/blast.pl#ppy-mir-645) |
| [MI0005235](http://www.mirbase.org/cgi-bin/mirna_entry.pl?acc=MI0005235) | [osa-MIR812c](http://www.mirbase.org/cgi-bin/mirna_entry.pl?acc=MI0005235) | 2 | 47 | 40 | 86 | + | 105 | 0.044 | [Align](http://www.mirbase.org/cgi-bin/blast.pl#osa-MIR812c) |
| [MI0005235](http://www.mirbase.org/cgi-bin/mirna_entry.pl?acc=MI0005235) | [osa-MIR812c](http://www.mirbase.org/cgi-bin/mirna_entry.pl?acc=MI0005235) | 135 | 180 | 40 | 86 | - | 105 | 0.044 | [Align](http://www.mirbase.org/cgi-bin/blast.pl#osa-MIR812c) |
| [MI0008296](http://www.mirbase.org/cgi-bin/mirna_entry.pl?acc=MI0008296) | [osa-MIR812g](http://www.mirbase.org/cgi-bin/mirna_entry.pl?acc=MI0008296) | 2 | 38 | 80 | 116 | + | 104 | 0.053 | [Align](http://www.mirbase.org/cgi-bin/blast.pl#osa-MIR812g) |

| **Accession** | **ID** | **Query start** | **Query end** | **Subject start** | **Subject end** | **Strand** | **Score** | **Evalue** | **Alignment** |
| --- | --- | --- | --- | --- | --- | --- | --- | --- | --- |
| [MIMAT0007220](http://www.mirbase.org/cgi-bin/mirna_entry.pl?acc=MIMAT0007220) | [oan-miR-138*](http://www.mirbase.org/cgi-bin/mirna_entry.pl?acc=MIMAT0007220) | 106 | 120 | 6 | 20 | - | 75 | 0.62 | [Align](http://www.mirbase.org/cgi-bin/blast.pl#oan-miR-138*) |
| [MIMAT0021758](http://www.mirbase.org/cgi-bin/mirna_entry.pl?acc=MIMAT0021758) | [aca-miR-138-1*](http://www.mirbase.org/cgi-bin/mirna_entry.pl?acc=MIMAT0021758) | 106 | 120 | 6 | 20 | - | 75 | 0.62 | [Align](http://www.mirbase.org/cgi-bin/blast.pl#aca-miR-138-1*) |
| [MIMAT0004607](http://www.mirbase.org/cgi-bin/mirna_entry.pl?acc=MIMAT0004607) | [hsa-miR-138-1-3p](http://www.mirbase.org/cgi-bin/mirna_entry.pl?acc=MIMAT0004607) | 106 | 121 | 5 | 20 | - | 71 | 1.3 | [Align](http://www.mirbase.org/cgi-bin/blast.pl#hsa-miR-138-1-3p) |
| [MIMAT0004668](http://www.mirbase.org/cgi-bin/mirna_entry.pl?acc=MIMAT0004668) | [mmu-miR-138-1-3p](http://www.mirbase.org/cgi-bin/mirna_entry.pl?acc=MIMAT0004668) | 106 | 121 | 7 | 22 | - | 71 | 1.3 | [Align](http://www.mirbase.org/cgi-bin/blast.pl#mmu-miR-138-1-3p) |
| [MIMAT0004734](http://www.mirbase.org/cgi-bin/mirna_entry.pl?acc=MIMAT0004734) | [rno-miR-138-1*](http://www.mirbase.org/cgi-bin/mirna_entry.pl?acc=MIMAT0004734) | 106 | 121 | 7 | 22 | - | 71 | 1.3 | [Align](http://www.mirbase.org/cgi-bin/blast.pl#rno-miR-138-1*) |
| [MIMAT0021759](http://www.mirbase.org/cgi-bin/mirna_entry.pl?acc=MIMAT0021759) | [aca-miR-138-2*](http://www.mirbase.org/cgi-bin/mirna_entry.pl?acc=MIMAT0021759) | 106 | 121 | 7 | 22 | - | 71 | 1.3 | [Align](http://www.mirbase.org/cgi-bin/blast.pl#aca-miR-138-2*) |

### Figure S3 The TE3 inserted into exon 3 may contain some microRNA sequences. The results were obtained by searching the inserted TE3 against sequences of mature miRNA in [www.mirBase.org](http://www.mirBase.org).

**
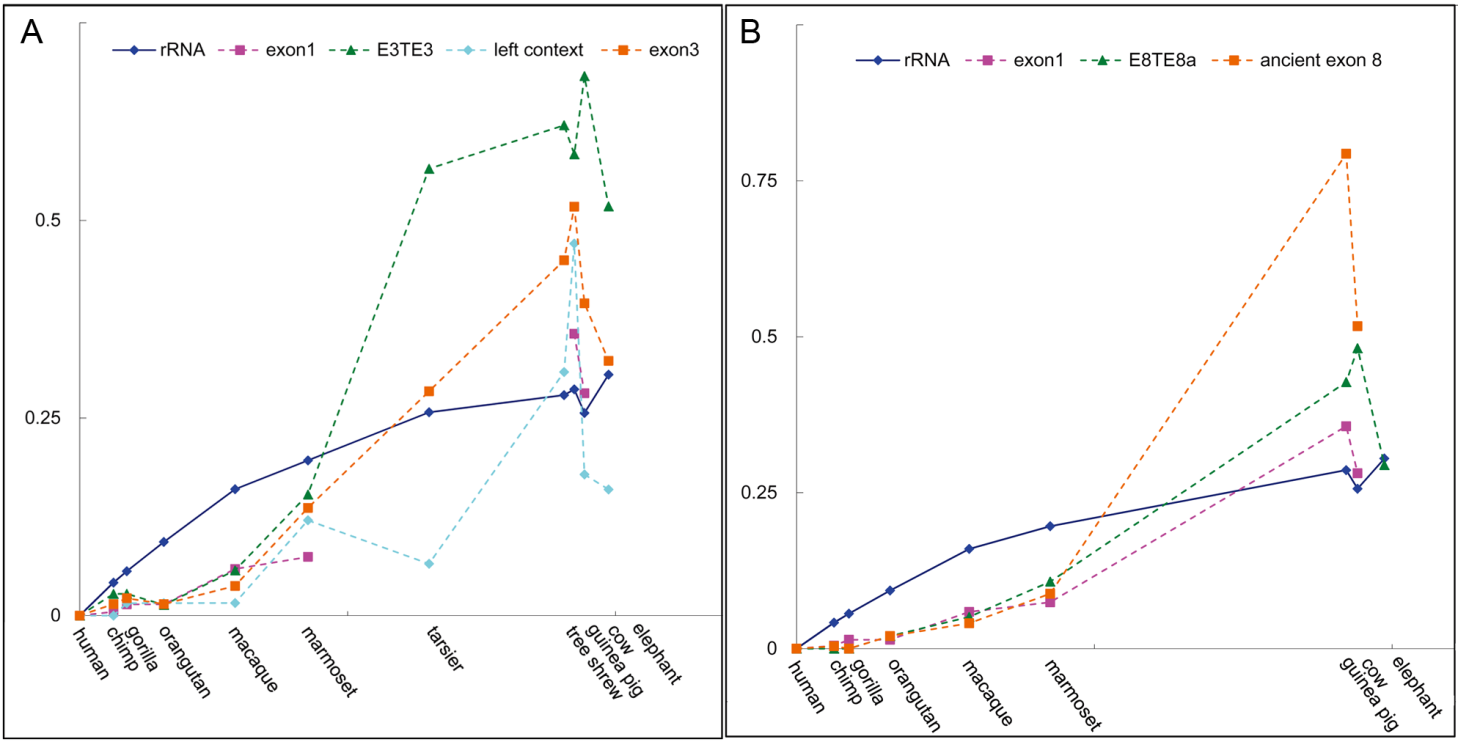
**

**Figure S4 The insertion of TE3 and TE8b into exon 3 and exon 8 affected the evolution of exon3 and exon 8.** The divergences between human and chimpanzee, gorilla, orangutan, macaque, marmoset, tarsier, three shrew, guinea pig, cow, and elephant are 6.3, 8.8, 15.7, 29.0, 42.6, 65.2, 90.4, 92.3, 94.2, and 98.7 Mya (the species divergence times were acquired from www.timetree.org). The displayed are the pairwise sequence distances between human and these species at these time points along the time axis. These pairwise distances indicate that exon 3 and exon 8 became conserved in simians after the insertion of TE3 and TE8b. (A) Pairwise distances of the concatenated 12S and 16S mitochondrial rRNAs, exon 1, E3TE3, the left context of the TE3 insertion site, and exon 3. (B) Pairwise distances of the concatenated 12S and 16S mitochondrial rRNAs, exon 1, E8TE8a, and the ancient exon 8 (the 5’ end + E8TE8a).


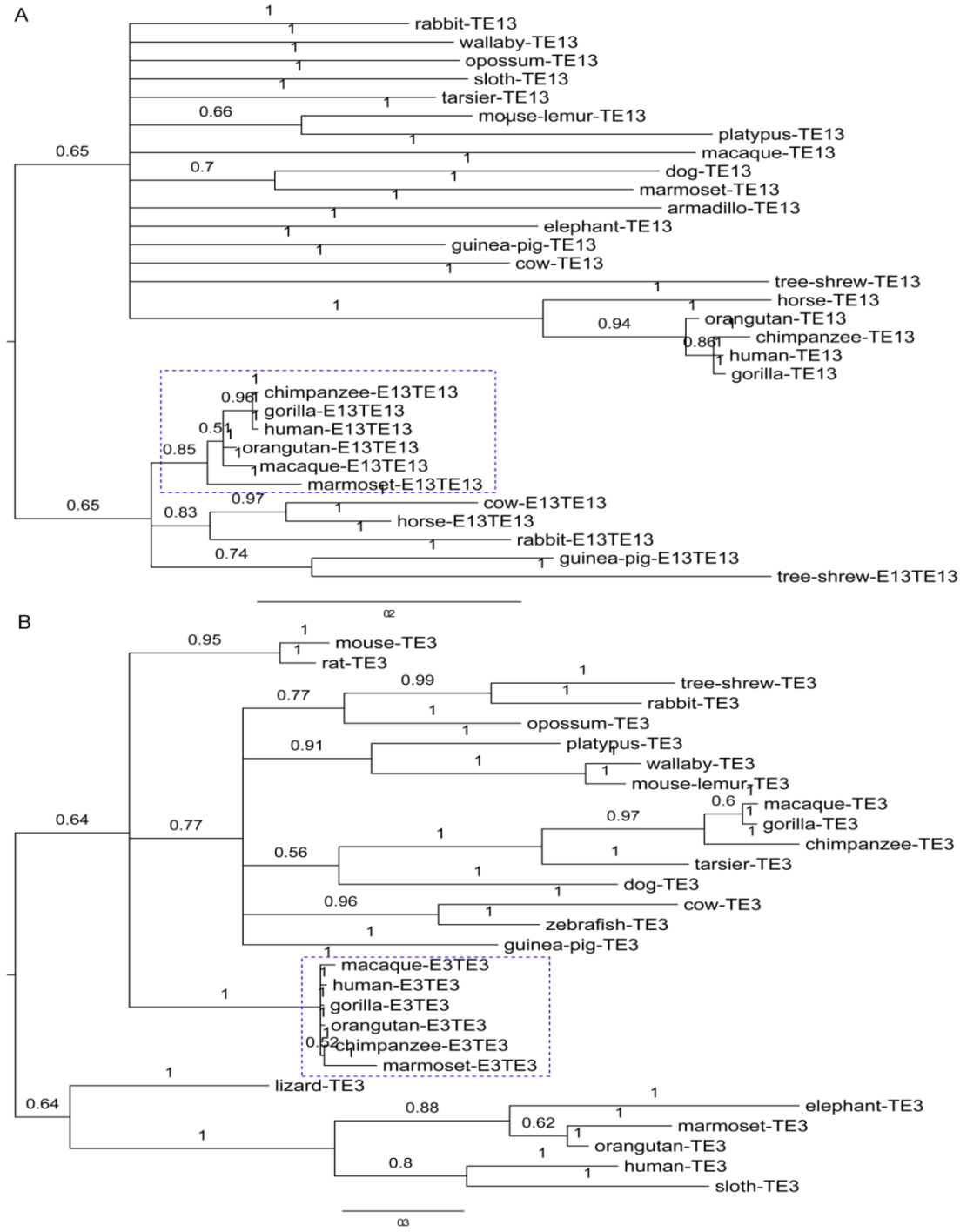


**Figure S5 Transposon sequences, after transforming or inserting into exons, have become conserved.** MEGA5.1 was used to predict the most appropriate substitution model (the Tamura 3-paramter + Γ model) and MrBayes was used to build the Bayesian trees. Numbers indicate posterior probabilities and the scale at the bottom measures genetic distances in nucleotide substitutions per site. (A) The tree of exon 13 and the highest-scoring free TE13. All exon 13 are grouped together, and in simians they have short exterior branches. (B) The tree of E3TE3 and the highest-scoring free TE3. All E3TE3 in simians are grouped together and have short exterior branches.


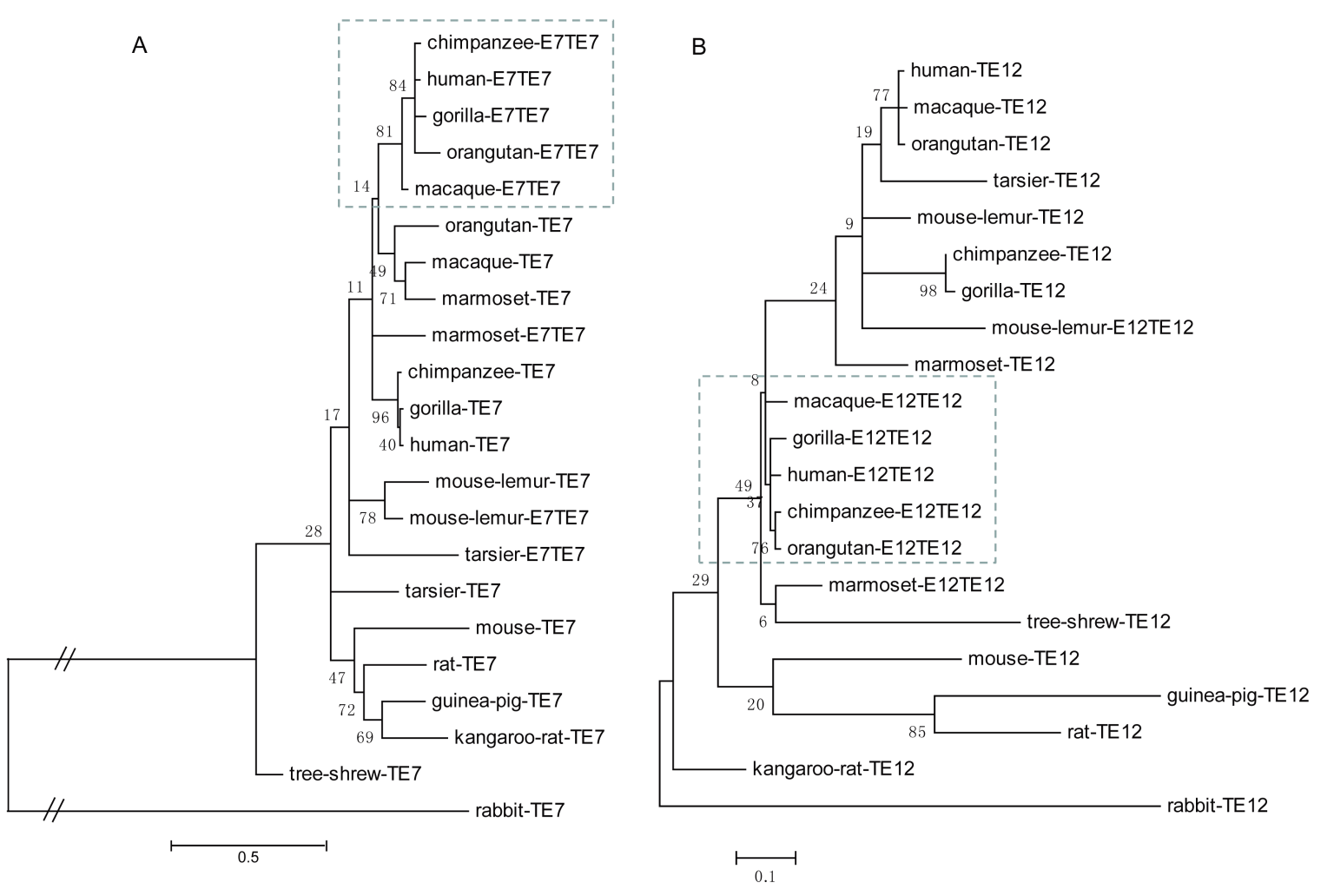


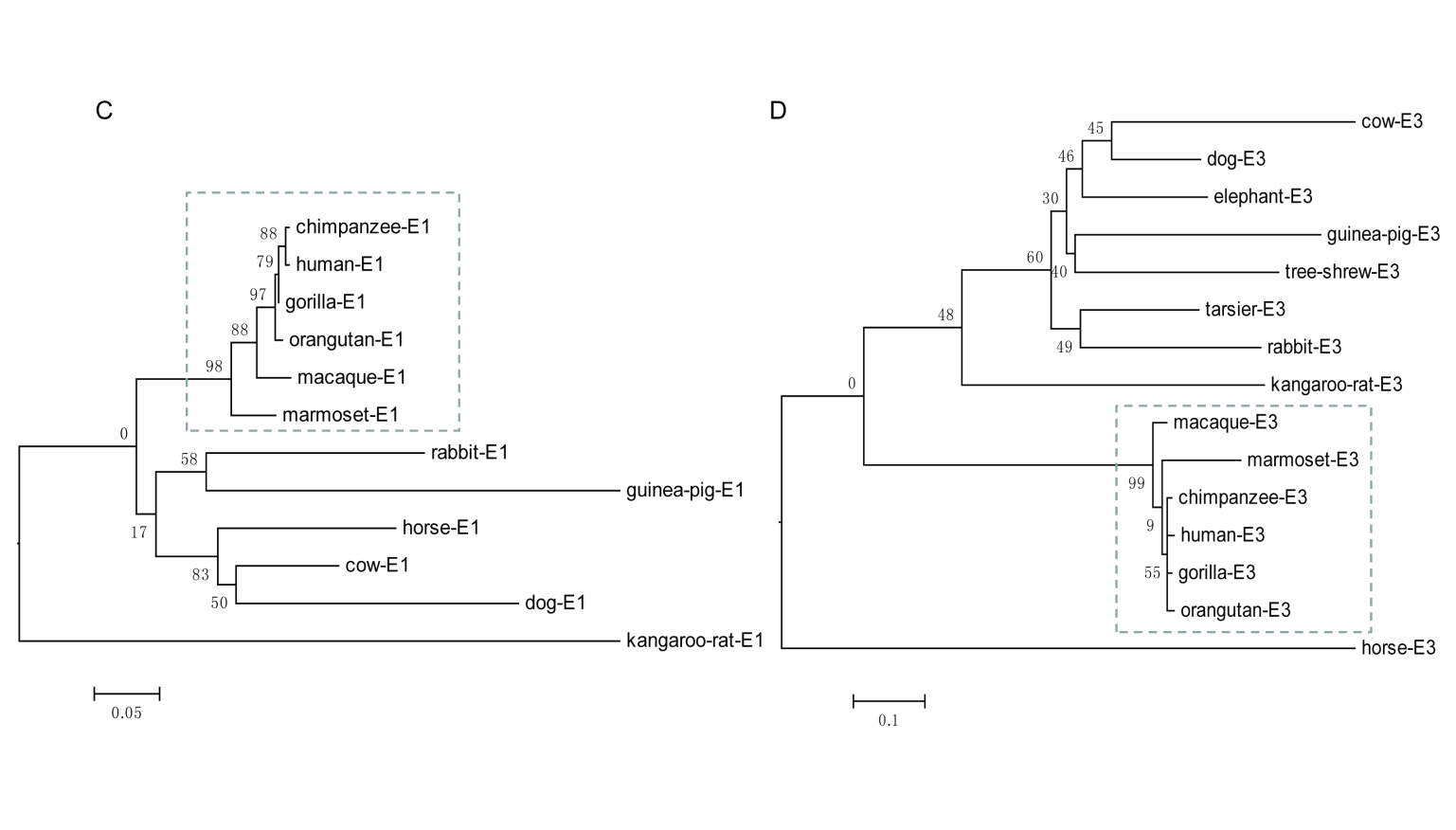


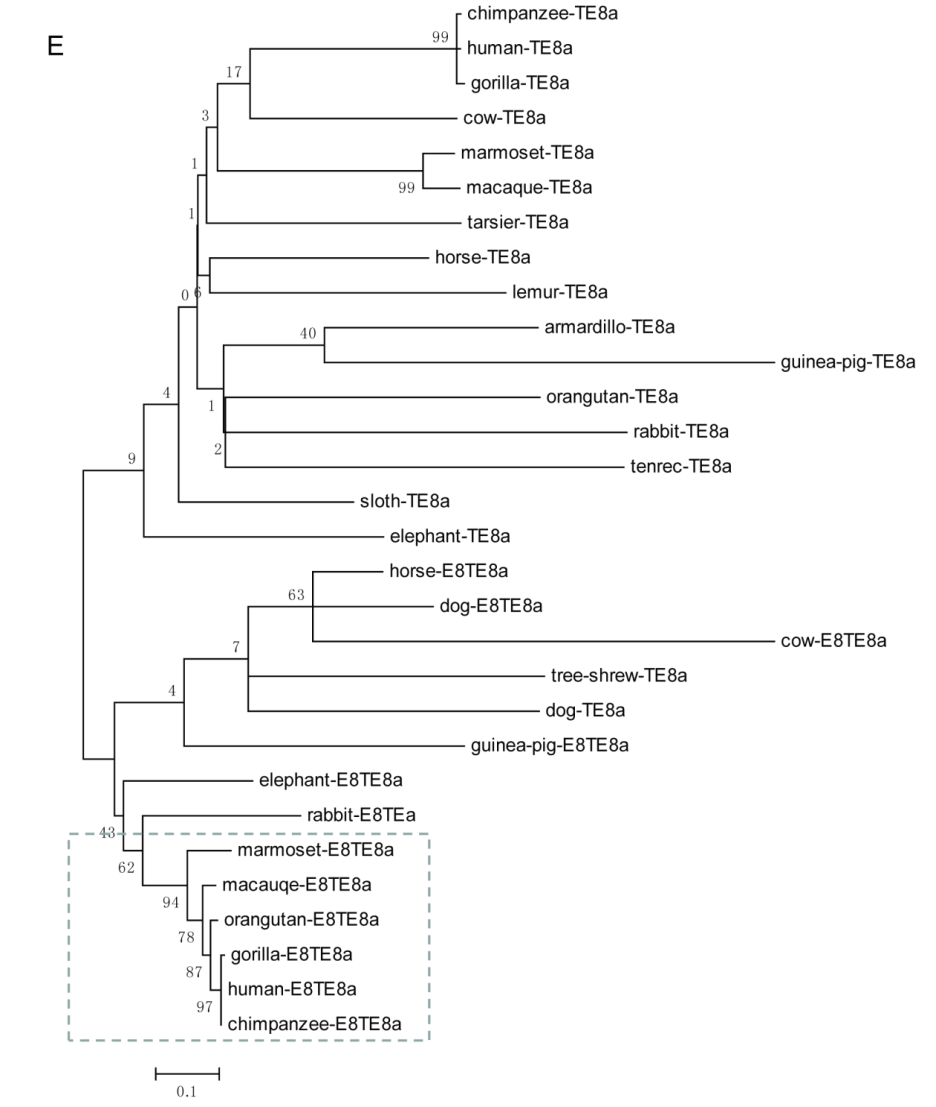


**Figure S6 Phylogenetic trees of exon 7/TE7, exon 12/TE12, exon 1, exon 3, and E8TE8a/TE8a.** Exon 7 and exon 12 are transposon-transformed exons, and E8TE8a is the inserted TE8a in exon 8. MEGA5.1 was used to predict the most appropriate substitution models and to build ML trees. Numbers indicate bootstrap values and the scale at the bottom measures genetic distances in nucleotide substitutions per site. (A) The ML tree of exon 7 (E7TE7) and the highest-scoring free TE7 based on the Kimura 2-parameter + Γ model. Exon 7 in simians are grouped together and have short exterior branches. (B) The ML tree of exon 12 (E12TE12) and the highest-scoring free TE12 based on the Kimura 2-parameter + Γ model. E12TE12 in simians are grouped together and have short exterior branches. (C) The ML tree of exon1 based on the Tamura 3-paramter + Γ model. It agrees with the species tree very well. (D) The ML tree of exon 3 based on the Tamura 3-paramter + Γ model. Compared with the tree of exon 1, it less agrees with the species tree. (E) The ML tree of E8TE8a and the highest-scoring free TE8a based on the Tamura 3-paramter + Γ model. Only E8TE8a in simians are reliably grouped together.


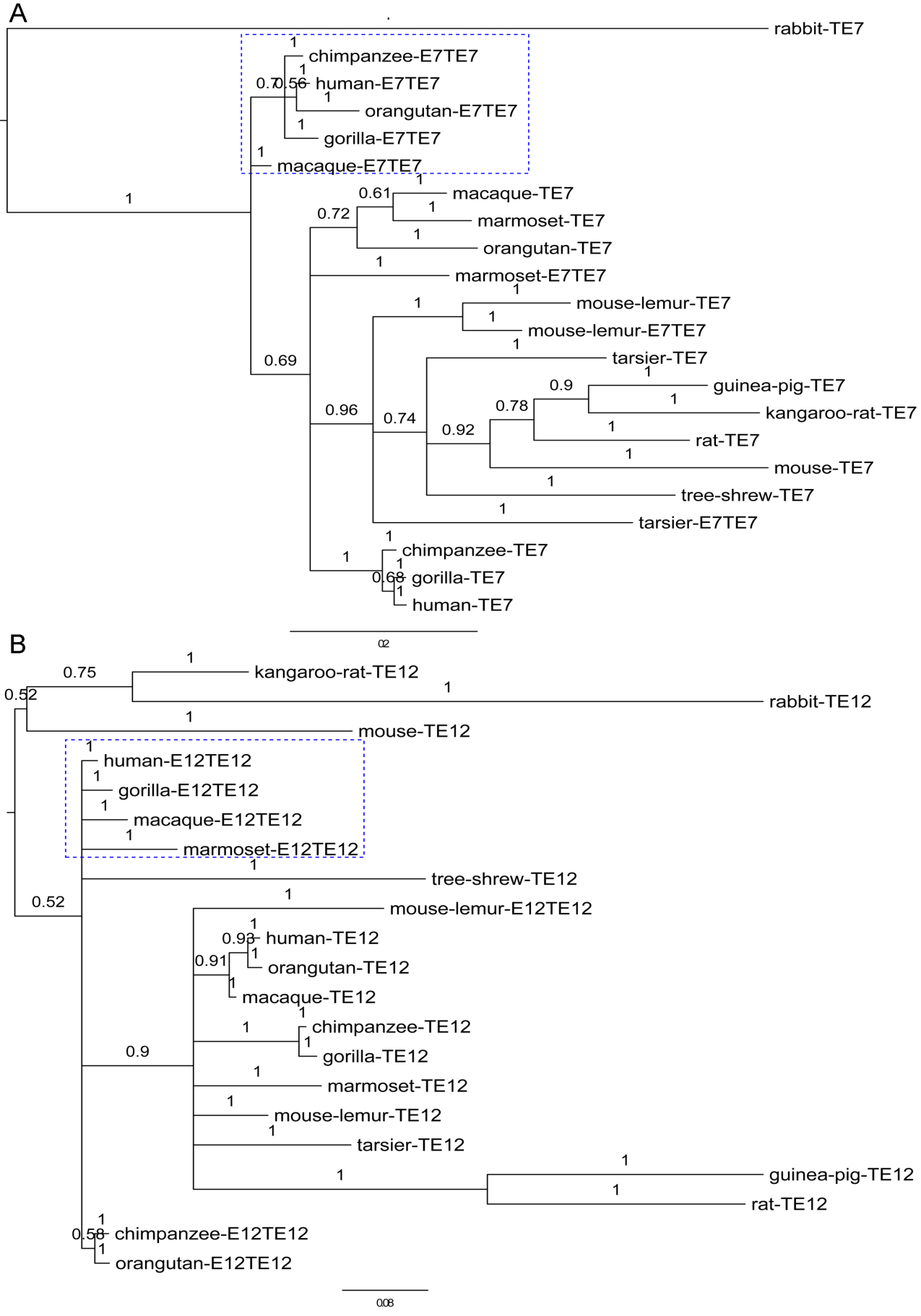


**
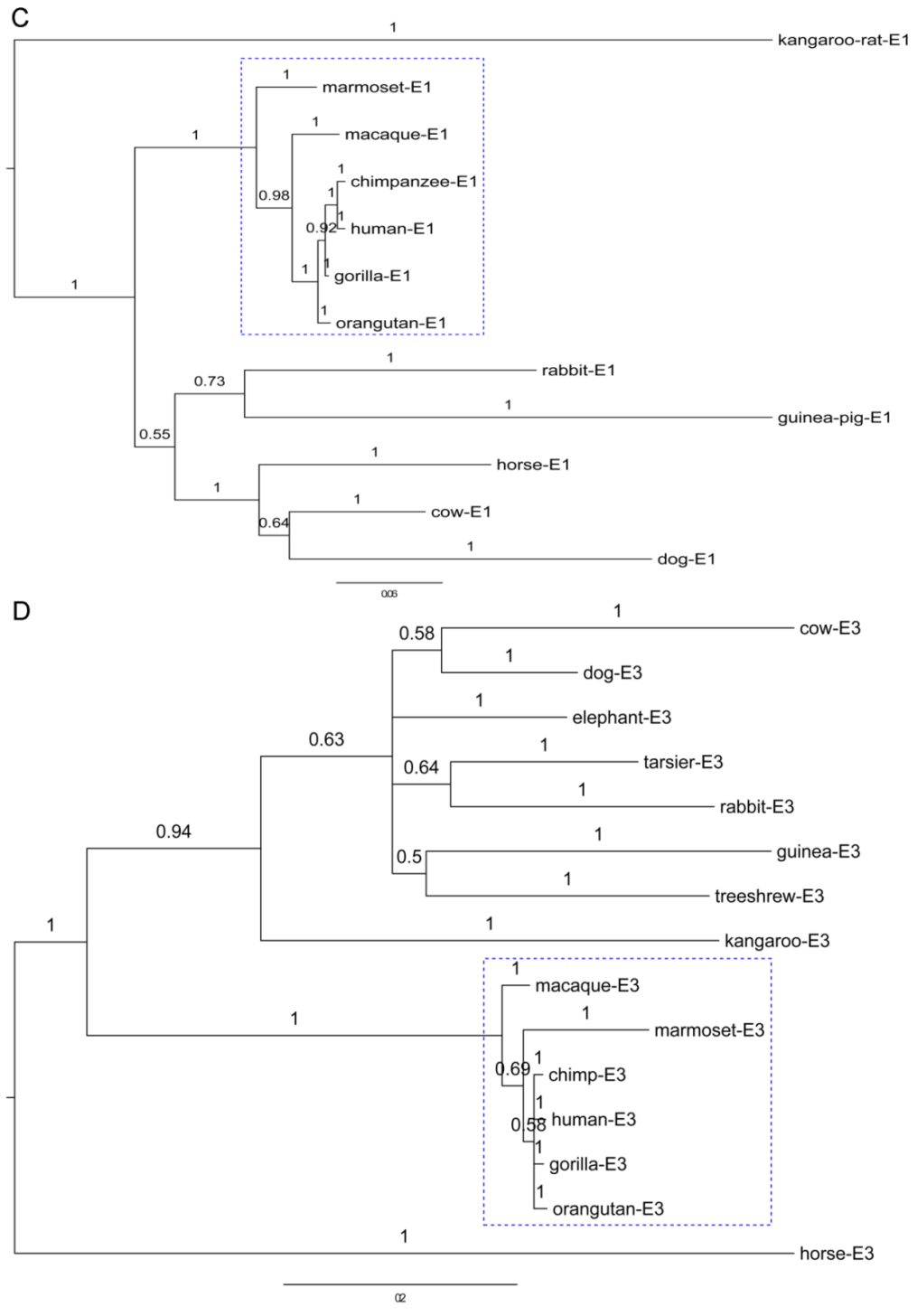
**

**
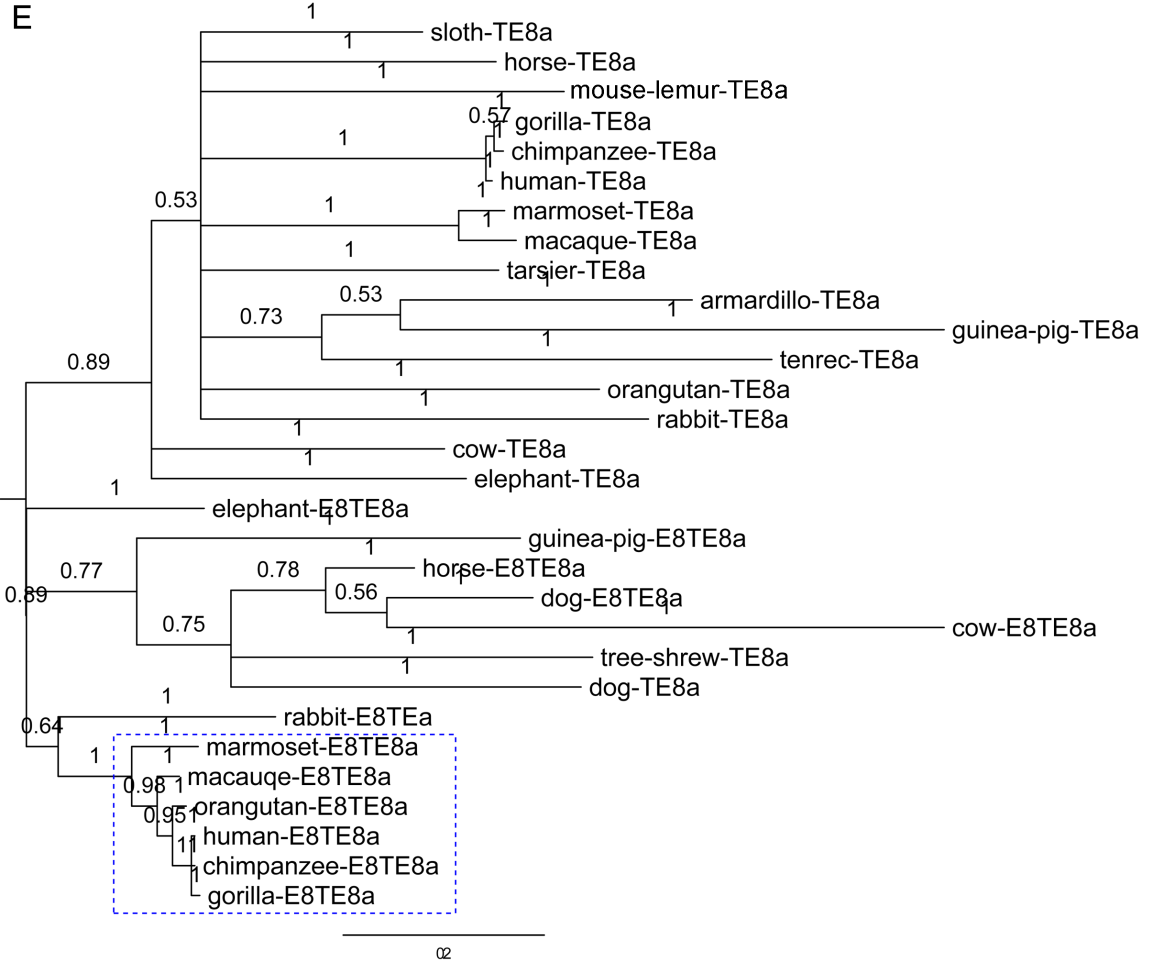
**

**Figure S7 Phylogenetic trees of exon 7/TE7, exon 12/TE12, exon 1, exon 3, and E8TE8a/TE8a.** Exon 7 and exon 12 are transposon-transformed exons, and E8TE8a is the inserted TE8a in exon 8. MEGA5.1 was used to predict the most appropriate substitution models and MrBayes was used to build Bayesian trees. Numbers indicate posterior probabilities and the scale at the bottom measures genetic distances in nucleotide substitutions per site. (A) The Bayesian tree of exon 7 (E7TE7) and the highest-scoring free TE7 based on the Kimura 2-parameter + Γ model. E7TE7 in simians are grouped together and have short exterior branches. (B) The Bayesian tree of exon 12 (E12TE12) and the highest-scoring free TE12 based on the Kimura 2-parameter + Γ model. E12TE12 in simians are grouped together and have short exterior branches. (C) The Bayesian tree of exon1 based on the Tamura 3-paramter + Γ model. (D) The Bayesian tree of exon 3 based on the Tamura 3-paramter + Γ model. Compared with the tree of exon 1, it less agrees with the species tree. (E) The Bayesian tree of E8TE8a and the highest-scoring free TE8a based on the Tamura 3-paramter + Γ model. Only E8TE8a in simians are reliably grouped together.

**Table S1 The occurrence of some identified transposons in other lncRNAs**

| **lncRNA** | **CM7 with Infernal scores** | **CM12 with Infernal scores** | **CM19 with Infernal scores** |
| --- | --- | --- | --- |
| BACE1AS_Human CB960709 | 113.26 |  |  |
| BACE1AS_Human BACE1-AS_RACE | 111.01 |  |  |
| DISC2_Human AF222981.1 | 96.34 | 40.75 |  |
| Emx2os_Human NR002791.2 | 74.50 | 53.38 |  |
| Kcnq1ot1_Human NR_002728.2 | 74.41 | 31.98 |  |
| linc1257_Mouse AK032971 | 37.65 |  |  |
| LUST_Human EF470865.1 | 21.70 | 56.99 |  |
| NEAT1_Human GQ859162.1 | 60.38 |  |  |
| NTT_Human U54776.1 |  | 56.40 |  |
| Otx2os1_Mouse NR_029384.1 |  |  | 35.55 |
| p53 Human NM_000546.4 | 66.82 | 43.17 |  |
| PRINS_Human NR_023388.1 | 36.78 |  |  |
| SNHG3_Human NR_036473.1 | 78.61 | 28.37 |  |
| SNHG4_Human NR_003141.3 |  | 37.23 |  |
| Xist_Human NR_001564.1 | 59.29 |  |  |
| SPRY4-IT1_Human AK024556.1 | 30.65 |  |  |
