# Supplementary material for: ANRIL/CDKN2B-AS shows two-stage clade-specific evolution and becomes conserved after transposon insertions in simians
Source: BMC Evol Biol. 2013 Nov 13;13:247. doi: 10.1186/1471-2148-13-247 (PMC3831594; doi:10.1186/1471-2148-13-247)
Supplement: Additional file 3 — ANRIL sequences in primates. [file 1471-2148-13-247-S3.pdf]

Supplementary file 3 – ANRIL sequences in primates

Chimpanzee ANRIL

AGCUACAUCCGUCACCUGACACGGCCCUACCAGGAACAGCCGCGCUCCCGCGGAUUCUGGUGCUGCUCGCGUCCCCGCUCCCCUAUUCCCCUUAUU
UUAUUCCUGGCUCCCCUCUUCGAAAGUCUUCCAUUCUUCAAACUAGAUUAUUUAAAAAUGAAAAAGGAAGAAAGGAAAGCGAGGUCAUCUCAUUGC
UCUAUCCGCCAAUCAGGAGGCUGAAUGUCAGUUUUGAACUAAAAGCCGCUCCGCUCCUCUUCUAGAUUUGGAAAACAAGCGAAAUUAAACUAAACC
GCUGCACGCCUCUGACGCGACAUCUGGACACGGCGCGGCGCUGGCGCUGCCGGAGCUGUCGGCCCGGCCUGGCGCCGGACUAGGACUAUUUGCCAU
GACAUUUCAAAGGAUUCCAAGAGAGAAUAUUGGUGUCCAUGCUAUGAUGAUUCCUCAGCUCCUCUCAUCUGAUCUCCGUCCUGGCCCCCAUGAUUU
UCUUUGUGGUAGUUAGGGUGUGGUAUGUGCCACUGAGGCCCACACCUAUUGCUGCAAUUUAUAGCACUGAUCUGUCAUCAAUACCACUUGCUGUCU
UGGAUGUGAAGAUGAUUUUUCCUGCAGGGAUUCCCUCUACAAAAUUAAAAACACUGGGCAUGAGGAAAUAAUAUUCAUGCUUUAAAUUGUCUUUUC
UCUUCACUACACCAGGGGUCCCCAGCCCNUAUGCCACAGANUGUGGCCNUAGUGUAGUGAAUAGAAAAGACAAUUUAAAGCAUGAAUAUUAUUUCC
UCGUGCCCAGUGUUUUUAAUUUUGUACUGGUCUGUGGCUUGUUAGAAACCAGGCUGCACAGCAGAAGGUGGGCAGCAGAUAUUGAGAAACCACAGA
AAG--AGAGAAGUUAAAUAAUUUUCCUGUCAAAGACCACAUCAAUGAUGAAGCCAGAAUUUGAGCUCAUUUACUUAACCACUGGACUACCUGCCUG
CCCUGUCGAGGAACUGCUAAGUGUCCCUUUUGAUGAGAAGAAUAAGCCUCAUUGUGAUUCAACAGCAGAGAUCAAAGAAAAGACUUCUGUUUUCUG
GCCACCAGAUAUAUGUUAUCUGUGCUUAAAGAAUUGAAAAACACACAUCAAAGGAGAAUUCUCUUGGAAAGAGAGGGUUCAAGCAUCACUGUUAGG
UGUGCUGGAAUCCUUUCCCGAGUCAGUACUGCUUUCUAGAAGAAAACCGGGGAGAUCUAUUUGGAAUGUAUCUAACUCCAAAGGAACCAUCAGAGG
UAACAGUAGAGACGGGGUUUCACCAUGUUGGCCAGACUGGUCUUGAACUCCCGACCUCGUGAUUCGCCCGCCUUGGCCUCCCAAAGUGCUGGGAUU
ACAGGUGUGAGACACCAUGCCCGGCGGAUAGAGAGAAUUUUGACAGUCUCUCCAAUGAACGCCUUCACUGAUAUCCAAAGCAUGAAGGACACACCA
GGGAAAAACAUAGACCUAACACAGGACAAAUGGAAUUAUUAGAAACAUUUUCUAGCAGAAGAACAAUAUUCUGUUGCCGUUUGAAUCUUUGCUUCU
UUCUACGUUUGACAAUGAGCCUAUCAUAUAAGCCCAAAUGUAAACAGAAAGAGGUUGAAUCAGUCACGAUAAGCCCAAUUAUGCUGUGGUAACAAA
CAACCUCAAAAUCUCAUUGGCUUAAAAUAUACAGAAUUAUUCUUACUCAUGGCACAUAUCCAUCUAUCAUCUGCAGGGGAUCUGCUCACUGAAGUC
ACUUAGGAACUUGGACUGAUGGAACGGCCACUUUUUGGUCACUAUAUGUAUUAAUCUGUUUUAAUCCUGCUGAUAAAGACCCAAAAUUGGGAACAA
AAAGAAGUUUAACUAGACUUACAGUUCCGCAUGGCUGAGGAGGCCUCAGAAUCAUGGUGGGAGGCGAAAGGCACUUCUUACAUGGUGGCAGCAAGA
GAAAAAUGAGGAAGAAGCAAAAGCGGAAACCUCUGAUAAACCCAUCAGAUCUCAUGAGACUUAUUCCACUAUCAAGAGAAUAGCAUGGGAAAGACU
GGCCCCCAUAAUUUACCUCCCUCUGGGUCCCUCCCUCAACAUGUGGGAAUUCUGGGAGAAAUAAUUCAAGAUAUGACACAUUCGUAAUUUAAACAG
AAGCCUACAAAGAACUCAUAAAUUGAAAGAAGAUAAUCUUUUCACAAGGUGAUGGAGGCUUUUUAUUUUGCCACAAAACCACUGGUGACGUUGCCU
GUGGCCACCUUGGAGAAGACACUGGAGGCCUGGGACAUGGAGACUGCUUUUCUGCAGAAACCACAUCCCUUGGAGUAAUGAGCUACACCUACCUCA
AUUAUUCAGUGCAGUACAACACUCCAGACAGAGUCUCACUCUGUCACCCAGGCUGGAGUGUAUUGGCAUGAUUACAGCUCACUGUGACCUUGAACU
CCCAGGCUCAAACCUGAGCAGCUGGGACUACAGGUGCACCACCAUGCAUGGUACCAGAGAUAUAAUAAUGAGAAACAGACAUGCUCCCUCCCCUCA
UUGAGGUUACAGCUUAGUGUGGAGACACACAGAUGCCUAACGCACUAUGGUAUGGAAGGUGCUAUGGACACAGUGCUCAAAUCCAUGAUCUACAUA
GGUGGAGAACUUCAGUUGAGGAAGUGGCAGGAAUUUGGGAAUGAGGAGCACAGUGAUUAAACUGGGGCCAUUCAUAUGAGAGUUUAAGAACUCAGA
CCAGUGACUUAGAUUGGCUUCUCUCACAUGGCAAGAAACAUUGCUGCUAGCACUUCCCGAGUUCUAUGUUCUACAACAUCCACCACUGGAUCUUAA
CAUAGACGUAAGAUCAAAUGUAAUAGCAUGUCAAACAAUGUGUAACUCCAGUUAUACAAACAUUACUGUAUCUCAUUGGGGAUACGAAGCUCUACA
CACUUGAAGAUGGUGAAGGAAUAUAAAAAUCUAUGUGUCUCACAGUCCAGACUUGGAGUACAAGUAAUAAGAAGAAUAAAACUUAAUCCCUCAAGU
AGAUUCACCAUAAGUUAGCUCAGAGCAAUUCCAGUGCAAGUAUGGUCUGUGAUCCAAUAGUAUCUUACAGACAGCAAGUUGAACAUUGUGGGAUGC
AUAAGCUAUUGAGGCCUUUGCAGCUUUCUGCUACAUGGAGGCUACGGCCAGAGUCAACAUUUAUGCUUUGCAGCACACUGGUCAGCUGUUUUUGCA
AAUCAGAUUAAAUAAUUUUUAAAUGAGGCUGAGAGCAUGGGAGAUACUAAUGUGUGUUUCCUUGUGAGCUACUGCAUAAGUUAGGAAAUUGAAAUA
CAGAAAGAUGAAAAGUGAUUUGCCCAAGUAUAUAGAUCAAAACUGUGGCAGAACCAGGACUGGAACCUAUAUCUCUCUACUAACGGUUUUUUUUAA
AAAAAACCUUGUUUCAAAAAUAUUAAAAAGUCACAAGAAAGGUAAACAUGUGAAUAAACAAAAUGAAGAAAAUAAAAAUUAUCCAGUAAUAACAUA
UUGGCAUAUGUCUUUCUGGUAUAUUUUCCUGUGUUGUCAUCAUUAUCAUCUCCAUCAUCAUUAUAUCCAUCAUUAUCAUCAUCAUCAUCAUCAuca
ccaUCAUCAUCAUCAUUAUCAUCACCAUAGCGAACACAUAAUGCUUACCUAGUGCCAGAUGCUGUCUAGGCAUUUUACAUGUGUUACUGGUAACUC
AUGUAAUCCUCAUAACAACCUUAUAAGGUGGUUGCUAUUAUCCCCAUGUUACAUAUGAAGAGACAGAAGCAUAAAGAAGUUGUACAGCUGGUAAUU
GGCUGGGAUGUGAACUUAAGCAGUCUAACCUUAGAGUAAUGAUUUUAACAACUAUGCUAUAUACAUACAAAUUUACAAAAUAAAACUGGGCUCAGA
CAAUAAA

Gorilla ANRIL

AGCUACAUCCGUCACCUGACACGGCCCUACCAGGAACAGCCGCGCUCCCGCGGAUUCUGGUGCUGCUCGCGUCCCCGCUCCCCUAUUCCCCUUAUU

UUAUUCCUGGCUCCCCUCGUCGAAAGUCUUCCAUUCUUCAAACUAGAUUAUUUAAAAAUGAAAAAGGAAGAAAGGAAAGCGAGGUCAUCUCAUUGC
UCUAUCCGCCAAUCAGGAGGCUGAAUGUCAGUUUUGAACUAAAAGCCGCUCCGCUCCUCUUCUAGAUUUGGAAAACAAGCGAAAUUAAACUAAACC
GCUGCACGCCUCUGACGCGACAUCUGGACACGGCGCGGCGCUGGCGCUGCCGGAGCUGCGGGCCCGGCCUGGCGCCGGACUAGGACUAUUUGCCAC
GACAUUUCAAAGGAUUCCAAGAGAGAAUAUUGGUGUCCAUGCUAUGAUGAUUCCUCAGCUCCUCUCAUCUGAUCUCCGUCCUGGCCCCCAUGACUU
UCUUUGUGGUAGUUAGGGUGUGGUAUGUGCCACUGAGGCCCACACCUAUUGCUGCAAUUUAUAGCACUGAUCUGUCAUCAAUACCACUUGCUGUCU
UGAAUGUGAAGAUGAUUUUUCCUGCAGGGAUUCCCUCUACAAAAUUAAAAACACUGGGCAUGAGGAAAUAAUAUUCAUGCUUUAAAUUGUCUUUUC
UCUUCACUACACCAGGGGUCCCCAACCCCUAGGCCACAGACUGUGGCCCUAGUGUAGUGAAUAGAAAAGACAAUUUAAAGCAUGAAUAUUUUUUCC
UCGUGCCAGUGUUUUUAAUUUUGUACUGGUCUGUGGCUUGUUAGAAACCAGGCUGCACAGCAGAAGGUGGGCAGCAGAUAUUGAGAAACCACAGAA
AGAGAGAAGUUAAAUAAUUUUCCUGUCAAAGACCACAUCAAUGAUGAAGCCAGAAUUUGAGCUCAUGUACUUAACCACUGGACUACCUGCCUGCCC
UGUCGAGGAACUGCUAAGUGUCCCUUUUGAUGAGAAGAAUAAGCCUCAUUCUGAUUCAACAGCAGAGAUCAAAGAAAAGACUUCUGUUUUCUGGCC
ACCAGAUAUAUGUUAUCUGUGCUUAAAGAAUUGAAAAACACACAUCAAAGGAGAAUUCUCUUGGAAAGAGAGGGUUCAAGCAUCACUGUUAGGUGU
GCUGGAAUCCUUUCCCGAGUCAGUACUGCUUUCUAGAAGAAAACCGGGGAGAUCUAUUUGGAAUGUAUCUAACUCCAAAGAAACCAUCAGAGGUAA
CAGUAGAGACUGGGUUUCACCAUGUUGGCCAGACUGGUCUUGAACUCCCGACCUCGUGAUUCACCCGCCUCGGCCUCCCAAAGUGCUGGGAUUACA
GGUGUGAGACACCACGCCCGGCAGAUAGAGAGAAUGUUGACAGUCUCUCCAAUGAACGCCUUCACUGAUAUCCAAAGCAUGAAGaGACACACCAGG
GAAAAACAUAGACCUAACACAGGACAAAUGGAAUUAUUAGAAACAUUUUCUAGCAGAAGAACACUAUUCUGUUGCCAUUUGAAUCUUUGCUUCUUU
CUAGGUUUGACAAUGAGCCUAUCAUAUAAGCCCAAAUGUAAACAGAAAGAGGUUGAAUCAGUCACGAUAAGCCCAAUUAUGCUGUGGUAACAAACA
ACCUCAAAAUCUCAUUGGCUUAAAAUAUACAGAAUUAUUCUUACUCAUGGCACAUAUCCAUCUAUCAUCUGCAGGGGAUCUGCUCACUGAAGUCAC
UUAGGAACUUGGACUGAUGGAAUGGCCACUUUUUGGUCACUAUAUGUAUUAAUCUGUUUUAAUCCUGCUGAUAAAGACCCAAAAUUGGGAACAAAA
AGAAGUUUAACUAGACUUACAGUUCCGCAUGGCUGAGGAGGCCUCAGAAUCAUGGUGGGAGGCGAAAGGCACUUCUUACAUGGUGGCAGCAAUAGA
AAAAUGAGGAAGAAGCAAAAGCGGAAACCUCUGAUAAACCCAUUAGAUCUAAUGAGACUUAUUUCACUAUCAAGAGAAUAGCAUGGGAAAGACCGG
CCCCUAUAAUUUACCUCCCUCUGGGUCCCUCCCUCAACGUGGGAAUUCUGGGAGAAACAAUUCAAGAUAUGACACAUUCAUAAUUUAAACAGAAGC
CUACAAAGAACUCAUAAAUUAAAAGAAGAUAAUCUUUUCACAAGGUGAUGGAUGCUUUUUAUUUUGCCACAAAACCACUGGUGACGUUGCCUGUGG
CCACCUUGGAGAUGACACUGGAGGCCUGGGACAUGGAGACUGCUUUUCUGCAGAAACCACAUCCCUUGGAGUAAUGAGCUACACCUACCUCAAUUA
UUCAGUGCAGUACAACACUCCAGACAGGGUCUCACUCUGUCAUCCCGGCUGGAGUGUAUUGGCAUGAUUACAGCUCACUGCGACCUUGAACUCCCA
GGCUCAAACCUGAGCAGCUGGGACUACAGGUGCACUACCAUGCAUGGUACCAGAGAUAUAAUAAUGAGAAACAGACAUGCUCCCUCCCCUCAUUGA
GGUUACAGCUUAGUGUGGAGACACACAGAUGCCUAACGCACUAUGGUAUGGAAGGUGCUAUGGACACAGUGCUCAAAUCCAUGAUCUACAUAGGUG
GAGAACUUCAGUAGAGGAAGUGGCAGGAAUUUGGGAAUGAGGAGCACAGUGAUUAAACUGGGGCUAUUCAUAUGAGAGUUUAAGAACUCAGACCAG
UGACUUAGAUUGGCUUCUCUCACAUGGCAAGAAACAUUGCUGCUAGCACUUCCCGAGUUCUACGUUCUACAACAUCCACCACUGGAUCUUAACAUA
GACGUAAGAUCAAAUGUAAUAGCAUGUCAAACAAUGUGUAACUCCAGUUAUACAAACAUUACUGUAUCUCAUUGGGGAUACGAAGCUCUACACACU
UGAAGAUGGUGAAGGAAUAUAAAAAUCUAUAUGUCUCACAGUCCAGACUUGGAGUACAAGUAAUAAGAAGAAUAAAACUUAAUCCCUUAAGUAGAU
UCACCAUAAGUUAGCUCAGAGCAAUUCCAGUGCAAGUAUGGUCUGUGAUCCAAUAGUAUCUUACAGACAGCAAGUUGAACACUGUGGGAUGCUUAA
GCUAUUGAGGCCUUUGCAGCUUUCUGCUACAUGGAGGCUAGGGCCAGAGUCAAGAUUUAUGCUUUGCAGCACACUGGUCAUCUGUUUUUGCAAAUC
AGAUUAAAUGAUUUGUAAAUGAGGCUGAGAGCAUGGGAGAUACUAAUGUGUGUUUCCUUGUGAGCUACUGCAUAAGUUAGGAAAUUGAAAUACAGA
AAGAUGAAAAUUGAUUUGCCCAAGCAUAUAGAUCAAAGCUGUGGCAGAACCAGGACUGGAACCUAUAUCUCUCUACUAACGGUUUUUUAAAAAAAA
UAACCUUGUUUCAAAAAUAUUAAAAAGUCACAAGAAAGGUAAACAUGUGGAUAAACAAAAUGAGGAAAAUAAAAAUUAUCCAGUAAUAACAUAUUG
GCAUACAUCUUUCUGGUAUAUUUCCCUGUGUUGUCAUUAUCAUCUCCAUCAUCAUUAUAUCCAUCAUUAUCAUCAUCAUCAUCgucACCAUCAUCA
UCAUCAUUAUCAUCACCAUAGCGAACACAUAAUGCUUACCUAGUGCCAGAUGCUGUCUAGGCAUUUUACAUGUGUUACUGGUAACUCAUGUAAUCC
UCAUAACAACCUUAUAAGGUGGUUGCUAUUAUCCCCAUGUUACAUAUGAAGAGACAGAAGCAUAAAGAAGUUGCACAGCUAGUAAUUGGCUGGGAU
UUGAACUUAAGCAGUCUAACCUUAGAGUAAUGAUUUUAACAACUAUGCUAUAUACAUACAAAUUUACAAAAUAAAACUGGGCUCAGACAAUAAA

Orangutan ANRIL

AGCUACAUCCGUCACCUGACACGGCCCUACCAGGAACAGCCGCGCUCCCGCGGAUUCUGGUGCUGCUCGCGUCCCCGCUCCCCUAUUCCCCUUAUU
UUAUUCCUGGCUCCCCUCGUCGAAAGUCUUCCAUUCUUCAAACUAGAUUAUUUAAAAAUGAAAAAGGAAGAAAGGAAAGCGAGGUCAUCUCAUUGC
UCUAUCCGCCAAUCAGGAGGCUGAAUGUCAGUUUUGAACUAAAAGCCGCUCCGCUCCUCUUCUAGAUUUGGAAAACAAGCGAAAUUAAACUAAACC
GCUGCACGCCUCUGAUGCGACAUCUGGACACUGCGUGGCGCUGGCGCUGCCGGAGCUGCGGGCCCGGCCUGGCGCCGGACUAGGACUAUUUGCCAC
GACAUUUCAAAGGAUUCCAAGAGAGAAUAUUGGUGUCCCUGCUAUGAUGAUUCCUCAGCUCCUCUCAUCUUAUCUCCAUCCUGGCCUCCAUGACUU
UCUuucuUUGUGGUAGUUAGGGUGUGGUAUGUG–CACUGAGGCCCACACUUACUGCUGCAAUUUAUAGCACUGAUCUGUCAUCAAUACCACUUGCU
GUCUUGGAUGUGAAGAUGAUUUUUCCUGUAGGGAUUCCCUCUACAAAAUUAAAAACACUGGGCAUGAGGAAAUAAUAUUCAUGCUUUAAAUUGUCU

UUUCCuuuaaauugucuuuucuCUUCACUACACCAGGGGUCCCCAACCCCUAGGCCACAGACUGUGGCCCUAGUGUAGUGAAGAGAAAAGACAAUU
UAAAGCAUGAAUAUUAUUUCCUGGUGCCCAGUGUUUUUAAUUUUGUACUGGUCUGUGGCUUGUUAGAAACCAGGCUGCACAGCAGAAGGUGGGCAG
CAGAUAUUGAGAAACCACAGAAAGAGAGAAGUUAAAUAAUUUUCCUGUCAAAGACCACAUCAAUGAUGAAGCCAGAAUUUGAGCUCGUGUACUUAA
CCACUGGACUGCCUGUCUGCCCUGUCAAGGAACUGCUAAGUCUCCCUUUUGAUGAGAAGAGUAAGUCUCAUUCUGAUUCAACAGCAGAGAUCAAAG
AAAAGACUUCUGUUUUCUGGCCACCAGAUAUAUGUUAUCUGUGCUUAAAGAACUGAAAAACACACAUCAAAGGAGAAUUCUCUUGGAAAGAGAGGG
UUCAAGCAUUAUUGUUAGGUGUGCUGGAAUCCUUUCCCGAGUCAGUACUGCUUUCUAGAACAGAACCGGGGAGAUCUAUUUGGAAUGUAUCUAACU
CCAAAGAAACCAUCAGAGGUAAUAGUGGAGACGGGGUUUCACCAUGUUGGCCAGACUGGUCUUGAACUCCAGACCUCAUGAUUCGCCUGCCUCCGC
CUCCCAAAGUGCUGGGAUUACAUGUGUGAGACACCACACUCAGCGGAUAGAGAGAAUUUUGACAGAGUCUCCcAAUGAAUGCCUUCACUGAUAUCC
AAAGCAUGAAGaGACACACCAGGGAAAAACAUAGACCUAACACAGGACAAAUGGAAUUAUUAGAAACAUUUUCUAGCAGAAGAACACUAUUCUGUU
GCCAUUUGAAUCUUUGCUUCUUUCUAGGUUUGACAAUGAGCCUAUCAUAUAAGCCCAAAUGUAAACAGAAAGAGGUUGAAUGAGUCACGAUAAACC
CAAUUAUGCUGUGGUAACAAACAACCUCAAAAUCUCAUUGGCUUAAAAUAUACAGAAUUAUUCUUACUCAUGGCACAUAUCCAUCAAUCAUCUGCA
GGGGAUCUGCUCACUGAAGUCACUUAGGAAUUUGGACUGAUGGAACUGCCACUUUUUGGUCACUAUAUGUAUUAAUCUAUUUCAUCCUGCUGAUA
AAGACCCAAAACUGGGAACAAAAAGAAGUUUAACUAGACUUACAGUUCCGCAUGGCUGAGGAGGCCUCAGAAUCAUGGUGGGAGGCGAAAGGCACU
UCUUACAUGGUGGCAGCAAGAGAAAAAUGAGGAAGAAGAAAAAGCGGAAACCUCUGGUAAACCCAUCAGAUCUCACGAGACUUAUUUCACUAUCAA
GAGAAUAGCAUGGAAAAGACCAACCCCCAUAAUUUAAUUACCUCCCUCUGGGUCCCUCCCUCAACAUGUGGGAAUUCUGGGAGAAACAAUUCAACG
CCUGGGACAUGGAUACUGCUUUUCUGCAGGAACCACAUCCCUUGGAGUAAUGAGCUACACCUACCUCAAUUAUUCAGUGCAGUACAACACUCCAGA
CAGGGUCUCACUCUGUCACCCAGGCUGGAGUGUAUUGGCAUGAUUACAGCUCACUGUGAUCUUGAACUCCCAGGCUCAAACCUGAGCAGCUGGGAC
UACAGGUGCACCACCAUGCAUGGUACCAGAGAUAUAAUAAUGAGAAACAGACAUGCUCCCUCCCCUCAUUGAGGUUACAGCUUAGUGUGGAGACAG
ACAGAUGCCUAAUGCACUAUAGUAUGGAAGGUGCUAUGGACACAGUGCUCAAAUCCAUGACCUACAUAGGUGGAGAACUUCAGUAAAGGAAGUGGC
AGGAAUUUGGGAAUGAAGAGCACAGUGAUUAAACUGGGGCCAUUCAUAUGAGAGUUUAAGAACUCAGGCCAGUGACUUAGAUUGGCUUCUCUCACA
UGGCAAGAAACAUUGCUGCUAGCACUUCUGGAGUUCUACGUUCUACAACAUCCACCACUGGAUCUUAACAUAGAUGUAAGAUCAAAUGUAAUAGCA
CGUCAAACAAUGUGUAACUCCAGUUAUACAAACAUUACUGUAUGUCAUUGGGGAUACGAAGCUCUACACACUUGAAGAUGGUGAAGGAAUAUAAAA
AUCUAUAUGUCUCACAGUCCAGACUUGGAGUACAAGUAAUAAGAAGAAUAAAACUUACUCCCUUAAGUAGAUUCACCAUAAGUUAGCUCAGGGCAA
UUCCAGUGCAAGUAUGGUCUGUGAUCCAACAGUAUCUCACAGACAGCAAGUUGAACAUUGUGGGAUGCAUAAGCUAUUGAGGCCUUUGAAGCUUUC
UGCUACAUGGAGGCUAGGGCCAGAGUCAAGAUUUAUGCUUUGCAGCACACUGGUCAGCUGUUUUUGCAAAUCAGAUUAAAUGAUUUUUAAAUGAGG
CUGAGAGCAUGGGAGAUACUAAUGUGUGUUUCCUUGUGAGCUACUGCAUAAGUUAGGAAAUUGAAAUACAGAAAGAUGAAAAGUGAUUCGCCCAAG
CAUAUAGAUCAAAGCUGUGGCAGAACCAGGACUGGAACCUAUAUCUCUCUACUAAUGUUUUUUUUUUUuucuAAAAAUAACCUUGUUUCAAAAAUA
UUAAAAAGUCACAAGAAAGGUAAACAUGUGGAUAAACAAAAUGAAGAAAAUAAAAAUUAUCCAGUAAUAACAUAUUGGCAUACGUCUUUCUGGUGU
AUUUUCCUGUGUUGUCAUUAUUAUCAUCUCCAUCAUCAUUAUAUCCAUCAUUAUCAUCAUCAACAUCAUCAccaUCAUCAUCAUCAUUAUCAUCAC
CAUAGCGAACACAUAAUGCUUACCUAGUGUCAGAUGCUGUCUAGGCAUUUUACAUGUAUUACUUGUAACCCAUGUAAUCCUCAUAACAACCUUAUA
AGGUGGUUGCUAUUAUCCCCAUGUUACAUAUGAAGAGACCGAAGCAUAAAGAAGUUGCACAGCUGGUAAUUGGCUGGGAUUUGAACUUAAGCAGUC
UAACCUUAGAGUAAUGAUUUUAACAACUAUGCUAUAUACAUACAAAUUUACAAAAUAAAACUGGGCUCAGACAAUAAA

Macaque ANRIL

AGCTACATCCGTCACCTGACACGGCCCTACCAGGAACAGCCGCGCTCCCGCGGGTTCTGGTGCTGCTCGCGTCCCCGCTCCCCTATTCCCCTTGCT
TTATTCCTAGCTCCCCTCGTCGAAAGTCTTACATTCTTCGAACTAGATTGTTTAAAAAAGCAAAAGGAAGAAAGGAAAGCGAGGTCATCTCATTGC
TCTCTCCGCCAATCAGGAGGCTGAATGTCAGTTTTGAACTAAAAGCCGCTCCGCTCCTCTTCTAGATTGGGAAAACAAGCGAAATTAAACGAAACC
GCTGCACGCCTCCGACGCGACATCTGGACACGGCGTGGCGCTGGCGCTGCGGGAGCTGCGGGCCCGGCCTGGCGCCGGACTAGTTCTATTTGCCAT
GACATTTCAAAGGATTCCAAGAGAGAATATTGGTGTCCATGCTGTGATGATTCCTCAGCTCCTCTCATCTGATCTCTGTCCTGGTTTCCATGACTT
TCTTTGTGGTAGTTAGGGTGTGATATGTGCCACTGAGGCCCACACCTATTGCTGCAATTTATAGCACTGATCTGTTATCAATACCACTTGCTGTCT
TGGATGTGAAGATGATTTTTCCTGCAGGGATTCCCTCTACAAAATTAAACACACTGGGCATGAGGAAATAATATTCATGCTTTAAATTGTCTTTTC
AATTCACTACACCAGGGGTCCCCAACCCCTAGGCCACAGACTGGCCCTGGTGTAATGAATAGAAAAGACAATTTAAAGCATGAATATTATTTCCTC
ATACCCAGTGTTTTTAATTTTGTACCGGGCTGTGACCTGTTAGAAACCAGGCTGCACAGCAGAAGGTGGGCAGCAGGTGAGCAAGCATTACTGCTT
GAGTTCCGCGTCCTGTCAGATCAGCAGCGGCATTAGATTCTCATAGGATATTGAGAAACCACAGAAAGATAGAGAAGTTAAATAATTTTCCTGTGA
AAGACCACATCAGTGATGAAGCCAGAATTTGAGCTCATGTACTTAACCATTGGACTACTTGCCTGCTCTGTCAAGGAACTGCTAAGTCTCCCTTTT
GATGAGAATAAGCCTCATTCTGATTCAACAGCAGAGATCAAAGAAAAGATTTCTGTTTTCTGGCCACCAGATATATGTTATCTGTGATTAAAGAAT
TGAAAAACACACATCAAAGGAGCATTCCCTTGGAAAGAGAGGGTTCAAGCATTATTGTTAGGTGTGCTGGAATCCTTTCCCAAGTCAGTACTGCTT
TCTAGAACAAAACCGGGAGATCCATTTGGAATGTATCTAACTCCAAAGAAACCATCAGAGGTAATAGTAGAGACGGGGTTTCACCATGTTGGCCAG

ACTGGTCTTGAACTCCTGACCTCATGATTTGCCCGCCTCAGCCTCCCAAAGTGCTGGGATTACAGGTGTGAGCCACCACGCCCGGCGGACAGAGAG
CATTTTGACAGTCTCTCCAATGAACACCTTCACTGATATCCAAAGCATGAAGACACACCAGGGAAAAACATGGACCTAACACAGGACAAATTGAAT
TATTAGAAACATTTTCTAGGAGAAGAACACTATTCTGTTGCCATTTGAATCTTTGCTTCTTTCTAGGTTTGACAATGAGCCTATCATACAAGCCCA
AATGTAAACAGAGAGAGGTTGAATCAGTCACGATAAACCCAGTTATGCTGTGGTAACAAACAACCTCAAAGTCTCACTGGCTTAAAATACACAGAA
TTATTCTTACTCATGGCACATATCCATCAATCATCTGCAGGGGATCTGCTCACTGAAGTCACTTAGGAACTTCGACTGATGGAACTGCCACTTTTT
GGTCGCTATATGTATTAATCTGTTTTCATGCTGCTGATAAAGACCCAAAACTGGGAACAAAAAGAAGTTTAACCAGACTTACAGTTCTGCATGGCT
AAGAAGGCCTCAGAATCATGGTGGGAGGCGAAAGGCACTTCTTATATGGTGGCAGCAAGAGAAAAATGAGGAAGAAGCAAAAGCAGAAACCTCTGC
TAAACCCATCAGATCTCATGAGACTTATTTCACTATCAAGAGAACAGCATGGGAAAGACCAGTCCCCATGAATTAATTACCTCTCCCTGGGTCCCT
CCCTCAACATGTGGGAATTCTGGGAGAAACAATTCAAGATATGACACATTCATAATTTAAACAAAAGCCTACAAAGAACTTATAAGTTGAAAGAAG
ATAATCTCTTCACAAGGTGATGGAGGCTTTTTATTTTGCCACAAAACCACTGCCGACGTTGCCTGTGGCCACCTTGGAGATGACACTGGAGGCCTG
GAACATGGAGACTGTTTTTCTGCAGAAACCACATCCCTTGGAGTAATGAGCTACACCTACCTCAATTATTCAGTGCAGTACAACACTCCAGACAGG
GCCTCTGTCTGTCACCCAGGCTGGAGTGTATTGGCATGATTACAGCTCACTGCGGCCTTGAACTCCCAGGCTAAAACCTGAGCAGCTGGGACTACA
GGTGCACCACCATGCATGGTACCAGAGATATAATAATGAGAAACAGACATGCTCCCTCCCCTCATTGAGGTTACAGCTTAGTGTGGAGACAGACAG
ATGCCTAATGCACCATAGTATGGAAGGTGCTATGGACACAGTGCTTAAATCCATAATCTACATAGGTGGAGAACTTCAGCAAAGGAAGTGGGAGTT
AGGAATTTGGGAATGAGGAGCACAGTGGGATTAAACTGGGGCCATTCATATGAGAGTTTAAGAACTCAGGTCAGTGACTTAGATTGGCTTCTCACA
CATGGCAGGAAACACTGCTGCTAGCACTTCCCGAGTTCTACATCCTACAACATCCACCACTGGATCTTAACGTAGATGTAAGATCAAATGTAATAG
CATGTCAAACAATGTGTAACTCAGTTATACAAACATTACTGTATCTCATTGGGGATACAAAGCTCTACACACTTGAAGGTGGTGAAGGAATATAAA
AATCTATATATCTCATGGTCCAGACTTGGAGTACAAGTAATAAGAAGAATAAAACTTAATCCCTTAAATAGATTCACCATAAGTTAGCTCAGGGCA
ATTCCAATACAAGTATGGTCTGTGATCCAACAGTATCTTACAGACAGCATGTTGAACATTGTGGGATGCATAAGCTATTGAGGCCTTTGAAGCTTT
CTACTACATGGAGGCTAGGGTCAGAGTCAAGATTTATGTTTTGCAGCACACTGGTCAGCTGTTTTTGCAAATCAGGTTAAATGATTTTTACATGAG
GCTGAGAGCATGGGAGATACTAATGTGTGTTCCCTAATGAGCTACTGCATAAGTTAGGAAATCGAAACACAGAAAGATGAAAAGTGATTTGCCCAA
GCCTATAGATAAAAGCAGTGGCAGAAACAGGACTGGAACCTATATCTCTCTACTAATTTTTTTTATTATAAAAATAATCTTGTTTCAAAATATTAA
GAAGTCACAACATATTGGCATATGTCTTTCTGGAATATTTTCCTGTGTTGTCATCATTATCATCTCCATCATCATTATATCCATCATCATCATCAT
CACCATCATCATCGTCATCATCATTATCATCACCATAGCCAACACATAATGCTTACCTAGTGCCAGATGCTGTCTAGGCATTTTACATATATTACT
GGTAACTCATGTAATCCTTATAACAACCTTATAAGGTGGTTGCTATTTTCCCCATGTTACATATGAAGAGACAGAAGCATAAAGAAGTTGTACAGC
TGGTAATTGGCTGGGATTTGAACTTAAGCAGTCTAACCTTAGAGTAACGATTTTAACAACTACACTATATACATACAAATTTACAAAATAAAACTG
GGCTGAGACAATAAA

Marmoset ANRIL

CGCUACAUCCGUCACCUGACACGGCCCUACCAGGAACCGCCGCGCUCCGGCGGGUUCUGGGGCUGCUCGCGUCCCCGCUCCCUCAUUCCCCUUGUU
UUAUUCCUGCCUCCCCUCGUCGAAAGUCUUCCAUUCUUCGAACUAGAUUAUUUAGAAGAGAAAAAGGAAGAAAGGAAAGCGAGGUCAUCUCGUUGC
UGUAUCCGCCAAUCAGGAGGCUGAAUGUCAGUUUUGAACAAAAAGCCGCUCCGCUCCUCUUCUAGAUUUGGAAAACUAGCGAAAUUAAAUGAAACC
GCUGCACGCUUCUGACGCGACAUCUGGACACGGCGUGGCGCUGCUGGAGCUGCGGGCCCGGCCGGGCACCGGACUAGGACUAUUUUCCAGGACAUU
UCAAAGGAUUCCAAGAGAGAAUAUUGGUGUCCAUGCUAUGAUGAUUCCUCACCUCUUCCCAUCUGUUCUCUGACCUGGCCUGCAUGACUCUGUUUG
UGGUAGUUAGGGUGUGGUAUGUACCACUGAGGCCCACACCUAUUGCUUCAAUUUAUAGCACUGACCUGUCAUCAAUAGUACUUGCUGUCUUGGAUG
UGGAGAUAAUUUUUCCUGCCAGGAUUCCCUCUAUAAAAUUAAAAAaUACUGGGCACUAGGAAAUAAUAUUCAUGCUUUAAAUUGUCUUUUCUAUUC
ACUACACCAGAAGUCUCCAGCCCUUAGGACCCGGACUGUGGCCUUUGUGUAGUGACUAGAAAAGACAGUUUAAAGCAUGAAUAUUAUUUCCUUGUG
CUUAGUGUUUUUAAUUUUGUACAGGUCCAUGGCCUGUUAGAAACCAGGCUGCACAGCAGAAGGUGAGCAGCAGAUAUUGAGAAACCACAGACAGUU
UGAGAAGUUAAAUAAUUUUCCUGUCAAAGACCAUAUCAAUGGUGAAGAUGGAAUUUGAGCUUAUGUACUUAACCAUUGGACUACCUGCAUGCCCUG
UCAAGGAACUGCUAAGCCUUCCUUUUGAUGAGAAGAAAAAGCCUCAUUCUGAUUCAACAGCAGAGAUCAAAGAAAAGACUUCUGUUUGCUGGCCAU
CAGAUAUAUGUUAUCUGUGUUUAAAGAAUUGAAAAACACACAUCAAAGGAACAUUCUCUUGGAAAGAGAGGAUUCAAGCAUUAUUGUUAGGUAUGC
UGGAAUCCUUUCCCAAGUCAGAACUACUUUCUAGAACAGAACUGGGGAGACCUAUUUGGAAUGUAUCUAACUCCAAAGgaaaAAACCAUCAGAGGU
AACAGUAGAGAAAGGGUUUCACCAUGUUGGUCAGGCUGGUCUUGAACUCCUCACcuCAAGUGAUCUGCCUGCCUCAGCCUCCCAAAGUGCUGAGAU
UACAGGCGUGAGCCACCUUGUGUGGCCUGCCUGACUUUUAAAAAUCUCUCCAAUGAACACCUUCACUGAUAUCCAAAGUGUGAAGAuAGACACUAG
GGAAAAACAUGGACCUAACAAAGGACAAAUGGAAUUGUUAGAAACAUUUUCCAGAAGAACACUACUCUGUGGCCAUUUGAAUCUUUGCUUCUUUCU
AGGUUUGACAAUGAGGCUAUCAUAUAAGCCCAAAUGUAAACUGAAAGAGGUUGAAUCAGUCAGGAUAAACCCUAUUGUGCUGUGAUAACAAACAAC
CUCAAAAUUUCAUUGGCUUAAAAUACACAGAAUUAUUCUUACUCAUGAUACAUAUUCAUCAAUGAUCUGCAAGGGAUCUGCUCACUGAAGUCACUg
AAGuGAACUCCAACUGAUGGAACUGCCACUUUUUGGUCACUAUGUGUAUUAAUCUGUUUUCAUGCUGCUAUUAAAGAUCUGAAACUGGGAACAAAU

AGAGGUUUAAUUAGACUUACAGUUCCACAUGGCUGAGGAGACCUCAGAAUCAUAAUGGAAGGUAAAAGGCACUUCUUACAUGGUGGGAGCAAGAGG
AAAAUGAAGAAGAAGCAAAAGUGGAAACCCCUGAUAAGCACAUCAGAUCUCGUGAGACUUAUUUCACUAUCAUGAGAGUAGCAUGGGAAAGACUGG
CUCCUAUGAUUCAAUUACCUCCCCCGGGUCCCUCCCACAAUAUAUGGGAAUUCUGGGAGAUACAAUUCAAGGUAUGACACAUUUAUAAUUUAAACA
AAAGuCCUACAAAGAACUCAUAAGUUAAAAUAAGACAAUCUCUUCAAAAGGUCAUGGAGGCUUCUUAUUUUGCCACAAAACCACUGGUGAUGUUGC
CUGUGGCCACCUUGGAGAUGACUCUGGAGACCUGGCACAUGGAGACUGCUUUUCUGUAGAAACCAUAUCCCUUGGUGUAAUGGGCUACACCUAUCU
CAAUUAUUCAGUGUAGUACAAUACUCCUGACAGGGUCUCACUCUGUUGCUCAGGCUGGAAUGUAUUUGCAUGAUUAUAGCUCACUACAGCCUUGAA
CUCCCAGGCUCAAACCUAAGCAGCUGGAACUAAAGGUGUACCACCAUGCAUGGUGCCAGAGAUAUUAUAAUUAGAAACAGACAUGCUCCCUCCCCU
CAUUGAGGUUACAGUUUAGUGUGGAGACAGACAGAUGCCUAAUGCACUAUAAUAUGGAAAGUGAUAUGGACACAGUGCUCCAAUUCAUGAUCUAUG
UAGGUGGAGAACUUCAGUAAAGGAAGCAGCAGUCAGGAAUUUGGGAAUGAGAAGCACAGUGAUUAAACUAGGGCCAUUCAUAUGGGAGUUUAAGAA
CUCUGGUCAGUGACUUAGAUUGGCUUCUCCCACAUGACAAGAAACAUUGCUGCCAGCACUUCCCAAGUUCUAUAUCCUACAACGUCUACCACUGGA
UCUatgttaAGUUCAAaAUGUAAUAGCAUGUCAAACAAUGUGCAACUC-AGUUAUACAAACAUUGCCAUAUCUCACUGGGGAUACGAAGUUCUACA
CACUUAAAAAUGGUAGAGGCAUAUAACAAUCUAUAUGUCUCACAGUUUAGACUUGGAGUAUAAGCAAUAAGAAUAAAUAUAGUCCCUUAAGUAUAU
UCACGUAAGUUAGCUCAGGGCAAUUCCAAUGCAAGUAUGAUCUGUGAUCCAACAGUAUCUUACAAAUAGCAAGUUGAACAUUGUGGGUUUCAUUAA
CUAUUGAGGACUUUGAAGCUUUCUGCUACAUGGAGGCUAGGGCCAGUGUCUAGAUUUAUACUUUGCAGCAUACUGGUCAGCUGUUUUUGCAAAUCA
GGUUAAAUAAUUUUUAAAUGAGGCUGAGAGCAUGGGAGAUAUUAAUGUAAGUUUCUUAAUGAGCUACUGCAUAUUUGAAACACAAAAAGAUCAAAA
GUGAUUUGACCAAGCAUAUAGAUCAAAGCUGUGGCAAAACCAGGAAUGGAACAUAUAUAUCUCUACUCAUGGUUUAAAACAAAAAUAAAACCUUGU
UUCAAAAAUAUUAAAAAGUCAUAAGAAAGGUAAACAUGUGGAUAAACAAAAUAAAAAAAAAGUUAUCCAGUAAUAACAUAUUGGUAUACAUCUUUC
U
